# Diagnostic and Therapeutic Role of Extracellular Vesicles in Articular Cartilage Lesions and Degenerative Joint Diseases

**DOI:** 10.3389/fbioe.2021.698614

**Published:** 2021-08-04

**Authors:** Kai Qiao, Qi Chen, Yiguo Cao, Jie Li, Gang Xu, Jiaqing Liu, Xiaolin Cui, Kang Tian, Weiguo Zhang

**Affiliations:** ^1^First Affiliated Hospital, Dalian Medical University, Dalian, China; ^2^Qingdao University of Science and Technology, Qingdao, China; ^3^Department of Orthopaedic Surgery and Musculoskeletal Medicine, University of Otago, Christchurch, New Zealand; ^4^Department of Medicine, Brigham and Women's Hospital, Harvard Medical School, Boston, MA, United States

**Keywords:** extracellular vesicle, cartilage lesions, degenerative joint disease, exosomes, cartilage repair

## Abstract

Two leading contributors to the global disability are cartilage lesions and degenerative joint diseases, which are characterized by the progressive cartilage destruction. Current clinical treatments often fail due to variable outcomes and an unsatisfactory long-term repair. Cell-based therapies were once considered as an effective solution because of their anti-inflammatory and immunosuppression characteristics as well as their differentiation capacity to regenerate the damaged tissue. However, stem cell-based therapies have inherent limitations, such as a high tumorigenicity risk, a low retention, and an engraftment rate, as well as strict regulatory requirements, which result in an underwhelming therapeutic effect. Therefore, the non-stem cell-based therapy has gained its popularity in recent years. Extracellular vesicles (EVs), in particular, like the paracrine factors secreted by stem cells, have been proven to play a role in mediating the biological functions of target cells, and can achieve the therapeutic effect similar to stem cells in cartilage tissue engineering. Therefore, a comprehensive review of the therapeutic role of EVs in cartilage lesions and degenerative joint diseases can be discussed both in terms of time and favorability. In this review, we summarized the physiological environment of a joint and its pathological alteration after trauma and consequent changes in EVs, which are lacking in the current literature studies. In addition, we covered the potential working mechanism of EVs in the repair of the cartilage and the joint and also discussed the potential therapeutic applications of EVs in future clinical use.

## Introduction

Articular cartilage defect (ACD) is a common clinical disease, which can be induced by trauma, joint degeneration, infection, autoimmune diseases, and among other reasons. Due to its special zonal and avascular structure, articular cartilage has limited self-repair capacity (Ossendorf et al., 2007). Thus, without timely and adequate treatment, injuries will further lead to various complications and eventually result in the development of osteoarthritis (OA) (Buckwalter and Lane, 1997). Conservative treatments, such as weight loss, physiotherapy, and biologicals, only provide a temporary palliative relief and fail to restore the damaged cartilage tissue. However, conventional surgical cartilage repair approaches, such as arthroscopic debridement, bone marrow stimulation procedures, and graft repair technologies, are not capable of reversing the progression of OA development. Although arthroplasty has been proved to be effective, it is only the last option after the failure of conservative treatments. In recent years, stem cell therapies, especially the transplantation of mesenchymal stem cells (MSCs), have been gradually applied for the prevention and treatment of OA. However, almost all MSCs have their own limitations, such as the loss of their availability due to the increasing donor age, limited proliferation capacity during *in vitro* expansion (Siddappa et al., 2007), and strict regulatory requirements throughout the isolation, collection, storage, and transportation process (Zhang et al., 2016). In addition, the use of live cells results in unavoidable safety issues such as immune rejection (Zhang et al., 2013), tumorigenesis due to uncontrolled cell differentiation (Nori et al., 2015), and the inability to remove the transplanted cells in case of adverse reactions (Toh et al., 2017). The initial assumption of using MSCs for cartilage tissue repair is based on their differentiation capability into chondrocytes and osteoblasts (Pittenger et al., 1999) to directly replace the damaged cartilage. Interestingly, an increased amount of evidence has shown that the repair mechanisms of MSCs also take into account the paracrine effect to endogenously stimulate cartilage repair. In recent years, extracellular vesicles (EVs), being considered as the most important critical functional soluble factors secreted by MSCs have gain their popularity in the cartilage tissue engineering field (Meirelles et al., 2009).

Extracellular vesicles are lipid vesicles secreted by cells with a bilayer structure. Under the examination by an electron microscope, they are spherical- or cup-shaped with a size of 30–5,000 nm. A large number of experiments have confirmed that, under physiological or pathological conditions, EVs can be secreted by most cell types (including synovial fibroblasts and chondrocytes) in an internal environment of the joint, reflecting the pathological alterations of the joint disease, which can be used for early diagnosis (Zhao and Xu, 2018). In addition, owing to their enriched cargo components such as RNAs and proteins, EVs can mediate the intercellular communication and improve the cellular function (Vlassov et al., 2012; Colombo et al., 2014), which could be novel therapeutics for the treatment of the joint disease. Furthermore, due to their nanocharacteristics, low immunogenicity, and excellent biocompatibility, EVs have the potential of acting as carriers for the targeted delivery of bioactive molecules or drugs (Kibria et al., 2018).

Although EVs have demonstrated their tremendous potential for the diagnosis and treatment of the joint disease in a number of studies at different levels (several studies have shown that the EVs secreted by MSCs could promote cartilage tissue regeneration and prevent cartilage degeneration in patients with early OA Cosenza et al., 2017; Zhang et al., 2018), technical challenges including the expansion of cells *in vitro* to harvest a sufficient number of EVs, along with the separation and purification of EVs (Tian et al., 2020), impede the clinical translation of EV-based research. In addition, the lack of informative and comprehensive reviews for understanding the underlying mechanism of EVs as a diagnostic or a therapeutic tool for degenerative joint disease further limits the motivation and capability of clinicians to potentially incorporate EVs into their clinical practice. Although recent literature studies have provided an overall picture of the landscape in EV-based research for cartilage repair, systemic reviews clearly categorizing different types of EVs underlying the working mechanism and therapeutic roles in cartilage regeneration remain elusive. In addition, the physiological environment and pathological alterations of the joint in a cellular and molecular level are overlooked in most of the reviews although this aspect is important for readers to fully understand the biological significance of EVs in the joint. As a result, in this review, we discuss the clinical applications and barriers of EVs from the perspective of biogenesis, cargo, and characteristics, as well as isolation methods. By further describing the physiological environment of the joint and its pathological alterations after trauma, we discuss the consequent changes in EVs and their potential as a diagnostic tool to identify joint diseases. In addition, the underlying working mechanism of EVs in the repair of the cartilage and joint is covered and complemented with recent examples, further demonstrating the potential therapeutic applications of EVs in future clinical use. We believe that there is much advancement that can be made as EV-based research is further progressed, and hope that this review, with the introduction of the fundamentals of EVs biogenesis and their roles in mediating intercellular communications and in combination with their working mechanism will help to motivate future opportunities within the EVs-based therapy in the diagnosis and treatment of the joint disease.

## Extracellular Vesicle

### Biogenesis

Based on their size, biogenesis, and release pathways, EVs can be simply classified into exosomes (30–150 nm), microvesicles (MVs) (100–1,000 nm), and apoptotic bodies (1,000–5,000 nm) (Yekula et al., 2020).

Exosomes, which have been heavily investigated by majority of the researchers, are cup- or sphere-shaped with a density of 1.13–1.19 g/ml (Asghar et al., 2020). They are cytosol-rich vesicles produced by endocytosis. Firstly, after the fusion of cargoes with cell membrane, plasma membrane involution induces the creation of endosomes. During the maturation of the endosomes, the late endosomes germinate intraluminal vesicles (ILVs) to form multivesicular bodies (MVBs), which further fuse with the plasma membrane to release ILVs through exocytosis (Li Z. et al., 2018; Rilla et al., 2019; Zhang et al., 2020). The consequently secreted ILVs within the extracellular space are called exosomes.

**Figure 1:**
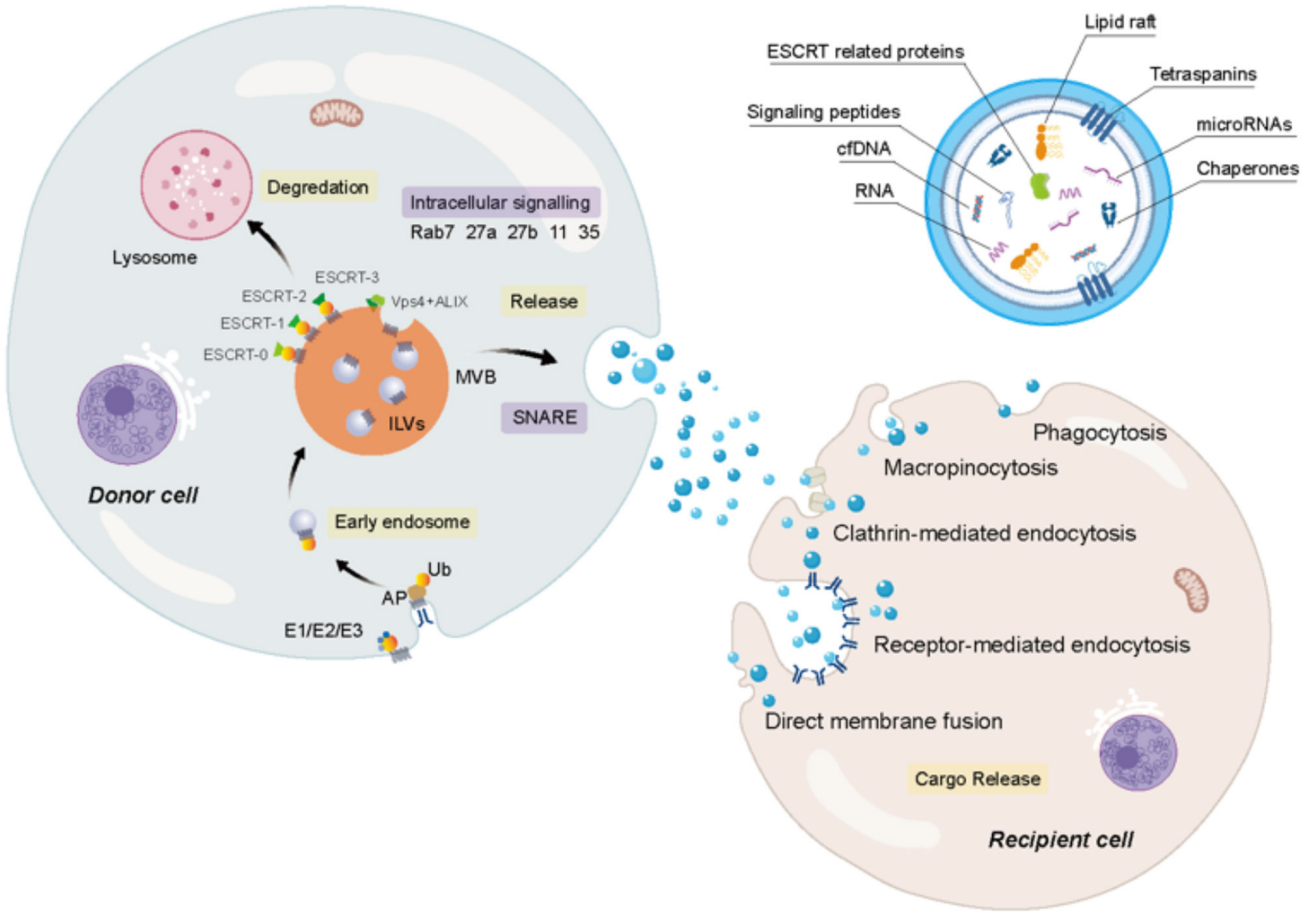
The biogenesis and secretion of extracellular vesicles (EVs) and the uptake of receptor cells. Firstly, after the fusion of particular cargoes with cell membranes, plasma membrane involution induces the creation of early endosomes. And then during the maturation of the endosome, the late endosomes germinate intraluminal vesicles (ILVs), forming multivesicular bodies (MVBs), which cannot only fuse with lysosomes for degradation, but also further fuse with plasma membrane to release exosomes. It is undeniable that the endosomal sorting complex required for the transport (ESCRT) complexes (ESCRT0, ESCRT-I, ESCRT-II, and ESCRT-III) are vital for MVB biogenesis. Several proteins, such as apoptosis-linked gene-2 (ALG-2) interacting protein X (ALIX), ATPase, and vacuolar protein sorting-associated protein (VPS4), also involve in regulating biogenesis, soluble N-ethylmaleimide-sensitive factor attachment protein receptors (SNAREs) participate in the fusion of MVBs with the plasma membrane. Members of the Rab family jointly regulate the secretion of exosomes, such as Rab-7, Rab-35, Rab-11, Rab-27a, and Rab-27b. The exosome contains a wide variety of cargoes. The uptake of exosomes can be inferred through several avenues.

The mechanisms of exosome biogenesis are highly complex, which involves a series of factors (Figure 1). To date, two pathways are mainly considered to involve in exosome biogenesis: the endosomal sorting complex required for the transport- (ESCRT-) dependent pathway and an ESCRT-independent pathway, both of which require the participation of ATP (Mianehsaz et al., 2019; Asghar et al., 2020). Some studies conclude that the ESCRT membrane-scission machinery consists of four-independent multi-protein subcomplexes, such as ESCRT0, ESCRT-I, ESCRT-II, and ESCRT-III (Hurley and Hanson, 2010; Wollert and Hurley, 2010). ESCRT-0, ESCRT-I, and ESCRT-II, the upstream complexes containing ubiquitin (Ub) and an ubiquitin binding domain (UBD), are important for interacting with Ub cargoes while ESCRT-III complex is vital for MVB biogenesis, virus germination, and cytokinesis and is responsible for the membrane division step of the membrane bud detachment from MVB lumen. According to a previous report, ESCRT-0 is considered as the main driving force for cargo accumulation (Hurley and Hanson, 2010; Wollert and Hurley, 2010). The direct interaction between Ub and the UBDs of ESCRT-0 mediates the upstream ESCRT complex containing UBD and Ub clustering. Although the incremental binding amount to Ub results in the increase of the size and the number of ESCRT-0-clustered domains on the membrane, they are not completely dependent on Ub, indicating that ESCRT-0 has the inherent ability to gather on the membrane (Wollert and Hurley, 2010). Intriguingly, it was found that there was no significant change in the number or characteristics of buds formed regardless of the presence of the upstream ESCRT-0 complex or downstream ESCRT-III vacuolar protein sorting-associated protein 20 (Vps20). This indicates that sprouting is an activity that only involves ESCRT-I and ESCRT-II complexes, where ESCRT-I- and ESCRT-II-mediated germination do not require the presence of cargo (Wollert and Hurley, 2010). ESCRT-I and ESCRT-II induce the formation of membrane buds and the confinement of cargo. The cargo in the buds can either spread laterally through the membrane or be effectively exchanged through the soluble phase. Physiologically low concentration of ESCRT-I and ESCRT-II can induce the membrane to germinate widely into the great unilamellar vesicles (GUVs) lumen but not into the lumen. In addition, ESCRT-I and ESCRT-II are located at the neck of the bud, indicating that they produce buds by stabilizing and inducing the bud neck (Hurley and Hanson, 2010; Wollert and Hurley, 2010). ESCRT-0, ESCRT-I, and ESCRT-II Ub domains were found to be colocalized with membrane buds, in which ESCRT-0 clusters the cargo and ESCRT-I and ESCRT-II generate membrane buds. Three subunits, Vps20, sucrose nonfermenting protein 7 (Snf7), and Vps24 of ESCRT-III, are important for the membrane division. The Vps20 subunit of ESCRT-III binds directly to ESCRT-II on the membrane and is activated by ESCRT-II on the membrane. While Vps20 does not exist on the membrane and inside the bud at all, it is confined to the neck because of its strong interaction with ESCRT-II (Hurley and Hanson, 2010; Wollert and Hurley, 2010). Snf7, a key cleavage factor, is thought to be an intermediate product of the pyrolysis reaction. In the scission reaction, Snf7 is located in the bud neck to prepare and separate the neck. During this reaction, the third subunit Vps24 that plays an important role in division starts to get involved, resulting in the occurrence of division. In short, ESCRT-0 is mainly responsible for cargo aggregation, and ESCRT-I and ESCRT-II supercomplex are responsible for membrane germination and cargo storage in buds. Meanwhile, ESCRT-III is responsible for scission (Hurley and Hanson, 2010; Wollert and Hurley, 2010). It has been reported that some proteins, such as apoptosis-linked gene-2- (ALG-2-) interacting protein X (ALIX), ATPase, and Vps4, also involve in regulating the ESCRT membrane-scission machinery (Mianehsaz et al., 2019). Interestingly, not all cells rely on the function of ESCRT for exosomal biogenesis and secretion. For example, lipids, such as sphingomyelinase (an enzyme that produces ceramide), sphingomyelin, ceramide, and sphingosine 1-phosphate, play a key role in an ESCRT-independent pathway and are mainly responsible for regulating the release of exosomes (Mianehsaz et al., 2019). Members of the Rab family jointly regulate the secretion of exosomes, such as Rab-35, Rab-11, Rab-27a, and Rab-27b (Asghar et al., 2020).

Microvesicles originate from the tightly packed cholesterol- and sphingolipid-rich membrane microdomains. In resting cells, negatively charged phosphatidylserine (PS) and phosphatidylethanolamine are separated in the inner membrane leaflet, whereas positively charged sphingomyelin and phosphatidylcholine are separated in the outer leaflet. The asymmetry of the membrane is maintained by the complementary action of two ATP-dependent transmembrane enzymes (called “flippase” and “floppase”). In the formation of MV due to a reaction to the inhibition of flippase activity and the activation of energy-dependent bidirectional transporter “scramblase,” phospholipids rapidly redistribute and randomly cross the bilayer, resulting in a gradual loss of membrane lipid asymmetry, which is considered as a signal to induce the membrane blistering and subsequent shedding of MVs exhibiting PS on their surface. The last key mechanism of MV formation is the cytoskeleton recombination process. This process involves the hydrolysis and destruction of proteins, which bind cytoskeleton elements to the plasma membrane, thus allowing vesicles to occur. The intracellular signaling events involved in this recombination vary greatly depending on the stimulation that triggers the MV release (activation or apoptosis). On one hand, the MV shedding from the stimulation of cells by physiological agonists commonly involves a calpain-dependent pathway. On the other hand, pro-apoptotic stimulation triggers the production of MV through a caspase-3-dependent pathway (Laberge et al., 2018).

Apoptotic bodies, different from the MVs and exosomes that are secreted during the normal cellular process, are released during programmed cell death. It is known that apoptosis is the main mechanism of cell death. In the process of apoptosis, the first step is nuclear chromatin pyknosis, the next step is the blistering of the cell membrane, and the final step is with the decomposition of the cell contents to form many different vesicles, namely apoptotic bodies. The existing data show that the blistering of membrane is partly mediated by an actin–myosin interaction. A few studies have also found that annexin V, thrombospondin (TSP), and C3b can be used as the three widely accepted markers of apoptotic bodies in the process of apoptotic body biogenesis, which indicate their role in mediating macrophage phagocytosis and the degradation of apoptotic bodies (Akers et al., 2013).

### Cargo and Characteristics

Extracellular vesicles have been identified to contain a variety of biomolecules, such as RNAs, DNAs, proteins, lipids, and metabolites, and are highly heterogeneous in terms of their size, cargo, source cell, and functional effect on receptor cells (Table 1). The cargoes present in EVs have been known to involve in intercellular communications *via* transmitting the signals to recipient cells (Teng and Fussenegger, 2020).

**Table 1:** Classification of extracellular vesicles (EVs).

**Type of vesicle**	**Exosomes**	**Microvesicles**	**Apoptotic bodies**
Biogenesis	Originate from MVBs, which further fuse with the plasma membrane	Formed by budding off the plasma membrane	Formed by the blistering of the cell membrane during programmed cell death
Size	30–150 nm	100–1,000 nm	1,000–5,000 nm
Nucleic acid	mRNA, miRNA, lncRNA, mtDNA	mRNA, miRNA, lncRNA, mtDNA	rRNA, DNA
Proteins (markers)	CD63, CD9, CD81, CD82, HSP70, HSP90, Tsg101, Alix/Syntenin	ARF6, VCAMP3	TSP, C3b
Lipids	Sphingomyelinase, sphingomyelin, ceramide	Cholesterol, sphingolipid	Phosphatidylserine

#### Nucleic Acid

A few studies have confirmed that EVs consist of many types of RNAs, such as microRNAs (miRNAs), messenger RNAs (mRNAs), transfer RNAs (tRNAs), small nucleolus RNAs (SnoRNAs), micronucleus RNAs (SnRNAs), Y-RNAs, vault-RNAs (vRNAs), long non-coding RNAs (LncRNAs), or ribosomal RNAs (RRNAs). In addition, mitochondrial DNA (mtDNA), single-stranded DNA (ssDNA), double-stranded DNA (dsDNA), and oncogene amplification products are also identified in exosomes (Bjorge et al., 2017; Mianehsaz et al., 2019; Asghar et al., 2020; Teng and Fussenegger, 2020). Although various RNA cargoes may be involved in epigenetic modification and changing of biological activity of cells, miRNA present in EVs is particularly important because of its capability to regulate the process of gene expression in receptor cells, resulting in physiological alteration. For example, miRNAs are involved in the regulation of matrix metalloproteinase (MMP), a disintegrin and metalloproteinase with thrombospondin motifs (ADAMTS), runt-related transcription factor (RUNX), type II collagen (COL-II) and various cytokines, which are crucial for the maintenance of articular chondrocyte ECM. Specifically, it has been noted that miR-140 has a protective effect and its expression is decreased in chondrocytes stimulated by OA and interleukin (IL)-1β (Asghar et al., 2020). Similarly, in the endotoxin injury model, the expression of inflammatory genes during the process of exogenous EVs delivery is enhanced and deregulated by the two key regulators of inflammation, miR-155 and miR-146, respectively (Alexander et al., 2015). In addition, many miRNAs derived from MSC EVs are the effective regulators of important signal transduction pathways, such as SMAD, protein kinase B (AKT), and extracellular signal-regulated kinase (ERK) pathways (Toh et al., 2017). Therefore, these miRNAs are likely to play a key role in mediating the efficacy of MSC EVs in the treatment of injuries and diseases such as OA. For example, miR-92a upregulates chondrocyte proliferation and matrix synthesis by targeting noggin3 through phosphatidylinositol-3-kinase/protein kinase B/the mammalian target of a Rapamycin (PI3K/AKT/mTOR) pathway, which mediates the role of MSC in reducing OA (Ning et al., 2013; Hou et al., 2015), while miR-23b plays a role in promoting the chondrogenic differentiation of human MSC by inhibiting a protein kinase A (PKA) signal pathway (Ham et al., 2012). In addition, miR-125b and miR320 can minimize the breakdown of extracellular matrix (ECM) by downregulating the expression of ADAMTS-4 (aggrecanase-1) and MMP-13, two ECM proteinases which are upregulated in human osteoarthritic chondrocytes (Toh et al., 2017).

#### Proteins

Extracellular vesicles also contain a wide range of proteins in addition to RNAs. The surface proteins of EVs have drawn a wide interest due to their role in identifying EVs and their subtypes. As mentioned previously, EVs can be divided into three categories based on their size and the formation mechanism, and those subtypes can be further identified through their unique surface makers. CD63 and CD9 can be the potential markers of exosomes. Other marker proteins in exosomes, such as Tetraspanins (CD81 and CD82), heat shock proteins (HSP70 and HSP90), tumor susceptibility gene 101 (Tsg101), and Alix/Syntenin, also play a role in the identification of exosomes. Recently proposed markers of MVs are ADP ribosylation factor 6 (ARF6) and vesicle-associated membrane protein 3 (VCAMP3). Meanwhile, TSP and C3b are recognized as apoptotic body markers. In addition, EVs also contain functional ubiquitous proteins, such as cytoplasmic proteins, flotillins, annexins, Rab proteins, and the molecules involved in signal transduction and metabolism. Vesicular proteins, reticular proteins, and transferrin receptors are also found in the cargo of EVs, playing an important role in the absorption of vesicles by recipient cells (Bjorge et al., 2017).

#### Lipids

Extracellular vesicles are also rich in lipids, and in different parent cells the content of lipids may vary. These lipids include sphingomyelin, PS, glycosphingolipid, phosphatidylethanolamine, phosphatidylcholine, cholesterol, phosphatidylinositol, ceramide, and diacylglycerol. The release and formation of exosomes are regulated by some lipid metabolic enzymes, such as phospholipase D2 (PLD2) or neutral sphingomyelinase (nSMase) (Bjorge et al., 2017; Mianehsaz et al., 2019). Those bioactive lipids cargo within EVs derived from stem cells have a variety of signal transduction functions, including immunomodulatory and anti-inflammatory activities (Bjorge et al., 2017). Those lipids are also a key component in the bilayer membrane structure of EVs. Due to the upregulation of sphingomyelin and cholesterol, the membranes of EVs are considered more stable compared to the cellular membrane with an improved resistance to physical or chemical destruction.

### Isolation

As a first step for all EVs-based research, the isolation and purification process of EVs are important (Li Z. et al., 2018). Common isolation techniques include ultracentrifugation (UC), size-exclusion chromatography (SEC), polymer precipitation, immuno-affinity purification, and microfluidics-based isolation techniques, and each technique has its own merits (Table 2).

**Table 2:** Methods of exosome separation.

**Method**	**Merit**	**Demerit**	**Example**	**References**
Ultracentrifugation	Most commonly used	Time-consuming, laborious, requires expensive equipment	Gurunathan et al. used ultracentrifugation and density gradient ultracentrifugation to isolate low-density and high-density vesicles in yeast, respectively.	Gurunathan et al., 2019
Size-based filtration, size-exclusion chromatography	Higher purity, fast	Contain the same size materials	For example, exosomes have been isolated from MSCs by size exclusion, and TEM analysis shows that they are intact.	Gurunathan et al., 2019
Polymer precipitation	Easy to operate, no require of expensive equipment	Exosomes of different sizes are mixed together	Niu et al. used polymer precipitation method to isolate EVs of endometrial cell line (Bolon et al., 2015).	Gurunathan et al., 2019
Immuno-affinity purification	Higher purity, fast, great for standard analysis method	Only separate exosomes with targeted proteins	Tauro et al. used immuno-affinity capture method to isolate exosomes from colon cancer cells, and the effect was better than that of ultracentrifugation and density gradient separation.	Gurunathan et al., 2019
Microfluidics-based isolation techniques	Higher yield and purity, shorter test time, low reagent consumption, small sample sizes, more cost-effective	Hard to use, requires expensive equipment	Kanwar et al. used the “ExoChip” platform to effectively capture EVs in circulation.	Gurunathan et al., 2019

#### Ultracentrifugation

Ultracentrifugation is the most commonly used technique for the isolation of EVs and a traditional method developed by Johnstone et al. to pellet the vesicles, which involves the separation of cells by centrifugation followed by the recentrifugation of the supernatant in 100,000 g for 90 min (Bjorge et al., 2017). This method is suitable for the granulation of lipoproteins, extracapsular protein complexes, aggregates, and other pollutants but not suitable for isolating exosomes for clinical samples because it is time-consuming and laborious. UC also requires expensive instruments and includes multiple complex centrifugation processes with multiple steps, potentially leading to contamination. Gurunathan et al. used UC and density gradient UC to isolate low- and high-density vesicles from yeast, respectively. Compared with UC, density gradient centrifugation has shown the purest extracellular body population (Hessvik and Llorente, 2018; Gurunathan et al., 2019).

The exosomes obtained by UC may contain heterogenic particles and potential pollutants. Due to a high centrifugation seep, the isolated exosomes may lose structural integrity, resulting in the reduction of quality and function loss. In addition, UC handling capacity is limited by the rotor capacity (Hessvik and Llorente, 2018).

#### Size-Based Filtration and SEC

Ultrafiltration (UF) is another popular method to separate exosomes according to size or molecular weight. Exosomes can be separated from other components in the sample using a membrane filter with the defined molecular weight or size exclusion limits. UF is faster than UC and does not require expensive equipment. High purity exosomes have been prepared by UF. Although pure vesicles can be obtained through this approach, the disadvantage is the difficulty to remove contaminated proteins. Filtration is often combined with UC in which cells and large EV are screened by membrane first, followed by the further separation of exosomes and proteins using UC (Gurunathan et al., 2019). Meanwhile, SEC technique on one hand can separate exosomes from proteins. On the other hand, it fails to separate exosomes from MVs, protein aggregates, liposomes, macromolecules, or particles (Hessvik and Llorente, 2018; Gurunathan et al., 2019). The separation of EVs according to size can also be achieved by using filters or chromatographic columns. Column chromatography allows a sequential elution of EV components of different sizes from a single column. For example, exosomes have been isolated from MSCs by size exclusion, and the transmission electron microscopy (TEM) analysis shows a great integrity of the purified exosomes (Gurunathan et al., 2019).

#### Polymer Precipitation

The precipitation method is used to separate exosomes by capturing and collecting exosomes of a certain size (50–150 nm) in the “polymer network” using a simple, fast, and low-speed centrifugation around 1,500 g. The method is easy with potential applicability to clinical use in large-scale production and does not need any expensive or special equipment. The polymers used to separate exosomes are made from non-toxic and inert materials that do not trigger any immune response *in vitro* or *in vivo*. The disadvantage of this method is that exosomes of different sizes are mixed together and non-exosomal substances, such as protein aggregates, may be co-isolated with exosomes. In addition, exosomes are potentially separated from proteins with polyethylene glycol, but not from MVs, protein aggregates, or liposomes (Gurunathan et al., 2019).

#### Immuno-Affinity Purification

The membranes of exosomes are known to contain large amounts of proteins. The immuno-affinity method is an approach to separate exosomes by using the interactions between proteins (antigens) and their antibodies through the specific interactions between the receptors and their ligands (Gurunathan et al., 2019). By using the immunoprecipitation technique of magnetic cell beads loaded with antibodies, this method can quantify the exosomes from antigen-presenting cells (Bjorge et al., 2017). Surface plasmon resonance (SPR) can also be used to analyze specific exosomal groups. SPR-based quantification of exosomes is based on capturing the exosomes on immune-functionalized surfaces and measuring the changes in refractive index caused by them. This method produces exciting results in the detection of exosomes containing specific exosomal markers such as CD63 and cancer-specific proteins (Hessvik and Llorente, 2018). The immuno-affinity method is fast, simple, and suitable for conventional laboratory equipment. This method uses the magnetic beads that are covalently coated with streptavidin, which can be coupled to any biotinylated capture antibody in a high-affinity manner. Tauro et al. used the immuno-affinity capture method to isolate the exosomes from colon cancer cells, and showed a better result in terms of the collection of the upregulated expressed exosome markers and associated proteins, compared to UC and density gradient separation (Bjorge et al., 2017). ELISA is another suitable immunoaffinity method for isolating the exosomes from body fluids, such as plasma and serum, using a variety of specific antibodies. To sum up, the immuno-affinity method using magnetic beads has higher capture efficiency and better sensitivity than other methods based on microwell plates because of its larger surface area and a homogeneous capture process (Gurunathan et al., 2019).

#### Microfluidics-Based Isolation Techniques

Due to technological progression, new technologies are constantly being developed. Traditional methods have a variety of shortcomings, such as low purity, long time period, high cost, low yield, and difficulty for standardization. In recent years, microfluidics-based technologies have gained their popularity for the separation and purification of exosomes based on the physical and biochemical properties of exosomes. In addition, being different from other separation approaches, this technique takes the advantage of innovative separation mechanisms such as acoustics, electrophoretic, and electromagnetic manipulations, as well as viscoelastic flow. The incorporation of immuno-affinity within the microfluidic chip further enhances the specificity and subtyping ability. Wang et al. have developed porous silicon nanowire-on-micropillar structures to distinguish the exosomes from all other EVs and cell fragments. The device preferentially captures exosomes of diameter between 40 and 100 nm while filtering out proteins, other EVs, and cell fragments. Kanwar et al. used the “ExoChip” platform to effectively capture EVs in circulation (Gurunathan et al., 2019). Compared to other approaches, a new separation method based on the microfluidic technology owns some advantages such as higher yield and purity, shorter test time, minimal reagent consumption, small sample sizes, and more cost-effective. Another significant advantage of the method is its compatibility with biological body fluids (Bjorge et al., 2017).

Taken together, the choice of separation methods will affect the studies into the results of exosome biogenesis and release obviously, so the best separation method should be suitable for the purpose of the study, downstream analysis, and which impurities are acceptable in this case.

## Physiological Environment of Joint

In order to properly use EVs for the diagnosis and treatment of the joint disease, it is important to understand the physiological environment of the joint.

**Figure 2:**
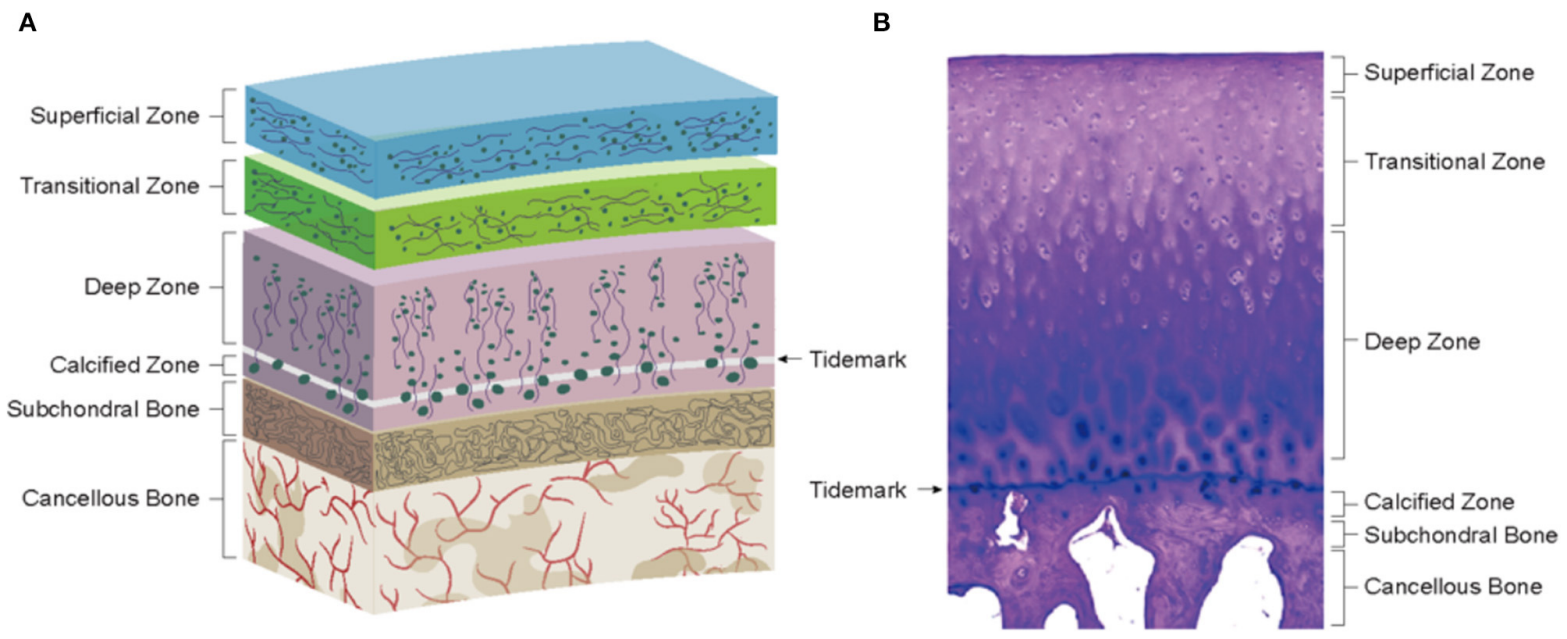
Physiological environment of healthy cartilage. **(A)** Schematics of cartilage zonal structure. **(B)** Histology of healthy cartilage (Loeser et al., 2012).

Articular cartilage is a layer of connective tissue covering the surface of the joint, which can lubricate the joint, reduce bone friction, and maximally absorb the vibration and impact caused by joint movement (Carballo et al., 2017). Articular cartilage is composed of chondrocytes and ECM. Chondrocytes are a kind of specialized mesenchymal cells whose main function is to produce and maintain their ECM. ECM of Chondrocytes often consists of water and a macromolecular framework construct consisting of collagens (mainly COL-II) and proteoglycans [mainly glycosaminoglycans (GAGs)]. The collagens are responsible for configuration and load-bearing and provide tensile strength for the cartilage tissue (Kuettner, 1992). The proteoglycan is extremely hydrophilic, making cartilage elastic and smooth and giving the necessary stiffness during the compression. Based on the biological differences of matrix composition and mechanical properties, articular cartilage can be divided into four zones: superficial zone, middle zone (transitional zone), deep zone (radial zone), and calcified cartilage zone (Figure 2). The first three zones can be collectively called hyaline cartilage zone. The calcified zone is tightly connected to the deep zone upward by a wave-shaped tidemark and anchored to the subchondral bone downward by an uneven comb-shaped cement line.

*Hyaline cartilage zone*: The distribution of chondrocytes was consistent with the trend of collagen fibrils. The superficial zone has flattened chondrocytes with collagen fibrils parallel to the joint surface, secreting lubricin. The collagen fibrils of the middle zone cross diagonally with spheroid chondrocytes (secreting COL-II and aggrecan) scattered among them. In the deep zone, collagen fibrils are mostly perpendicular to the articular surface, and chondrocytes are arranged in a columnar form (Becerra et al., 2010).

*Calcified cartilage zone*: This zone is the result of endochondral ossification (EO), and its tissues are characterized by obvious mineralization and low cell density (mostly round hypertrophic chondrocytes) (Hoemann et al., 2012). Being an interface between the cartilage and bone, the calcified cartilage zone plays an important role in osteochondral structure and function. Biologically, this zone plays a barrier role in inhibiting the invasion of blood vessels from the bone zone and preventing the mineralization of the hyaline cartilage zone. In terms of mechanics, the presence of tidemark and cement line not only facilitates stress dispersion but also converts the shear forces generated during joint movement into compressive and tensile stresses, preventing the cartilage from being torn off (Schinagl et al., 1997). Moreover, it has been shown that the calcified zone also has a role similar to a semi-permeable membrane, allowing the transmission of signaling substances (Arbabi et al., 2016). This allows the bone-cartilage junction to form an independent but integrated functional unit with an interaction through a cellular and molecular crosstalk due to the changes in the surrounding tissues, leading to structural and functional alterations. For example, osteoblasts and osteoclasts can interact with each other in an intercellular communication to maintain bone tissue homeostasis *in vivo*. Osteoblasts exert regulatory effects on osteoclasts through their production of receptor activator of nuclear factor-κB ligand (RANKL) and osteoprotegerin (OPG) (Bolon et al., 2015). RANKL interacts with RANK expressed in osteoclasts to activate osteoclasts while OPG inhibits the binding of RANKL to RANK. In addition, exosomes are also involved in the establishment and maintenance of bone remodeling. Sun et al. demonstrated that osteoclast-derived exosomes could transfer miR-214 into osteoblasts and thus inhibit its activity and interfere with bone formation (Sun et al., 2016), which is similar to the experimental results of Li et al. (2016). In another study, Chen et al. designed an alginate construct based on cartilage progenitor cells (CPCs) and found that chondrocyte-derived exosomes could increase intrastructural collagen deposition and reduce vascular ingrowth, promoting the formation of phenotypically stable cartilage structures, thus confirming the chondrogenic role of chondrocyte-derived exosomes (Chen et al., 2018). In addition, the cartilage and subchondral bone can interact with each other through signaling pathways, such as transforming growth factor-β (TGF-β) (van der Kraan et al., 2010) and Wnt/β-catenin (Lu et al., 2013), to affect the homeostasis of adjacent tissues.

## Changes in Evs to Reflect the Joint Disease

Given the fact that alterations in the microenvironment will lead to the changes in cellular behavior and that EVs are heavily involved in cellular communication, it is likely that the cargo of EVs will change with disease onset and progression. To fully understand the biological significance of EVs in the joint, a comprehensive illustration about the pathological alterations of OA and consequent changes in EVs is necessary.

### Pathological Alteration

Due to the impact of external forces, such as impact load or torsion load, together with its limited regenerative capacity, articular cartilage is prone to injury (Dhollander et al., 2012). A few studies have shown that cartilage or osteochondral damage occurs in up to 60% of the patients undergoing arthroscopic surgery (Becher et al., 2015). These lesions usually cannot be repaired on their own because of the poor self-repair capacity of cartilage, leading to chronic joint pain and reduced mobility, which can further induce the development of OA. The end-stage manifestations of post-traumatic OA are obtained similar to primary OA. However, the early pathological changes may differ.

#### Traumatic Cartilage Injury

When the articular surface is compressed in a physiological range, the load on the joint surface leads to the flow of fluid (a hydrated proteoglycan gel) within the matrix. The pressure is alleviated by the elastic deformation of the cartilage and is evenly distributed to the subchondral bone plate through a fibrillar meshwork, resulting in the reduction of local pressure and protection of the cartilage from mechanical damage (Martin et al., 2020). When the single or repetitive impact exceeds the maximal capacity of a matrix macromolecular framework, the fibrillar meshwork will be destroyed, leading to the locally abnormal pressure and damage to the subchondral bone. The specific manifestations are as follows:

**I**. When the joint is subjected to acute stress (violent extrusion and ligament tear), the microstructure, composition, and metabolism of cartilage will be changed, resulting in the reduction of chondrocytes, loss of cartilage matrix, and rupture of collagen (Loening et al., 2000).
**II**. When the joint is subjected to chronic or repetitive stress (long-term heavy exercise and high-load exercise), the severity of tissue damage increases with the increase of load and the number of loading cycles, which leads to progressive cartilage degeneration (Zimmerman et al., 1988). Based on the depth of the defect and the corresponding potential repair response, articular surface injuries can be further divided into three categories:
  i. *Cartilage degeneration*: The defect is limited to the superficial zone, with the microdamage to chondrocytes and ECM (cell clustering and matrix fibrillation) (Tiderius et al., 2005) and no significant mechanical damage to the cartilage surface. At the same time, chondrocytes can still sense the change of matrix composition and react by balancing anabolism and catabolism to supplement the loss of macromolecules in the process of degradation (Martin and Buckwalter, 2000). When the accumulated micro-damage becomes irreversible and the cells are unable to recover the matrix, the chondrocytes and their ECM will be exposed to overload and rapidly diminish.
  ii. *Cartilage rupture*: The defect involves the middle zone, deep zone, and calcified zone, and visual changes, such as chondral fissures, flap tears, or chondral defects, happen in the cartilage (Martin et al., 2020). Although there is reactive proliferation of cells at this time, the increase in mitotic activity is transient and the proliferated cells and newly synthesized matrix are not able to fill the tissue defect.
  iii. *Osteochondral fracture*: When the defect reaches the subchondral bone zone, it can trigger a restorative inflammatory response (Buckwalter et al., 2003), and also generate a new articular cartilage through the tissue of new granulation. However, the composition and structure of the cartilage tissue to be repaired are usually between hyaline cartilage and fibrocartilage, which fails to function as healthy cartilage.

#### Degenerative Cartilage Injury

In contrast to post-traumatic OA usually occurring in young people with induced trauma, primary OA mainly affects the elderly. With aging, the autophagy of chondrocytes gradually decreases, inducing senescence (Carames et al., 2010). By secreting senescence-associated secretory phenotypes (SASPs) such as pro-inflammatory cytokines, chemokines, and proteinases, senescent cells alter the tissue microenvironment and impair the repair capacity of the cartilage while promoting immune cells to participate in inflammatory response (Kuilman et al., 2010). For instance, IL-1 can increase the production of nitric oxide (NO), which blocks proteoglycan biosynthesis and induces chondrocyte apoptosis (Hedbom and Häuselmann, 2002) and also significantly enhance the expression of matrix degrading proteinases (mainly MMP-13), thereby inhibiting collagen production (Mengshol et al., 2000). Increased cell size, flattening, and vacuolization have been observed through microscopic histopathology (Kuilman et al., 2010).

Hypertrophy is also an important pathological change in OA chondrocytes. In the stressed environment, partial chondrocytes lose their differentiated phenotype and enter into an EO-like state of proliferation (which is likely responsible for cell clustering) with abnormal hypertrophic differentiation (Dreier, 2010). The hypertrophic chondrocytes express the genes associated with osteogenesis and produce the mineralized ECM proteins that calcify and stiffen the matrix, leading to the apoptosis of the chondrocytes due to the lack of access to nutrients, which results in lacunar emptying of the calcified cartilage zone and the subsequent formation of vascular channels in response to vascular endothelial growth factor (VEGF) (Sharif et al., 2004; Mackie et al., 2008; Findlay and Atkins, 2014; Charlier et al., 2016; Li and Dong, 2016). Microscopically, the vascular proliferation of the subchondral bone can be observed and invade into the cartilage area, causing tidemark duplication (Suri and Walsh, 2012).

An increase in senescence and hypertrophy of chondrocytes further leads to a reduction in anabolic signaling pathways and an increase in catabolic signaling pathways (Forsyth et al., 2005), These alterations downregulate the activity of chondrocytes and eventually lead to cartilage degeneration and the progressive loss of the hyaline cartilage zone, resulting in a direct bone-to-bone contact during motion, which triggers pain and aggravates the tissue damage and also the progression of OA.

### Changes in EVs

**Figure 3:**
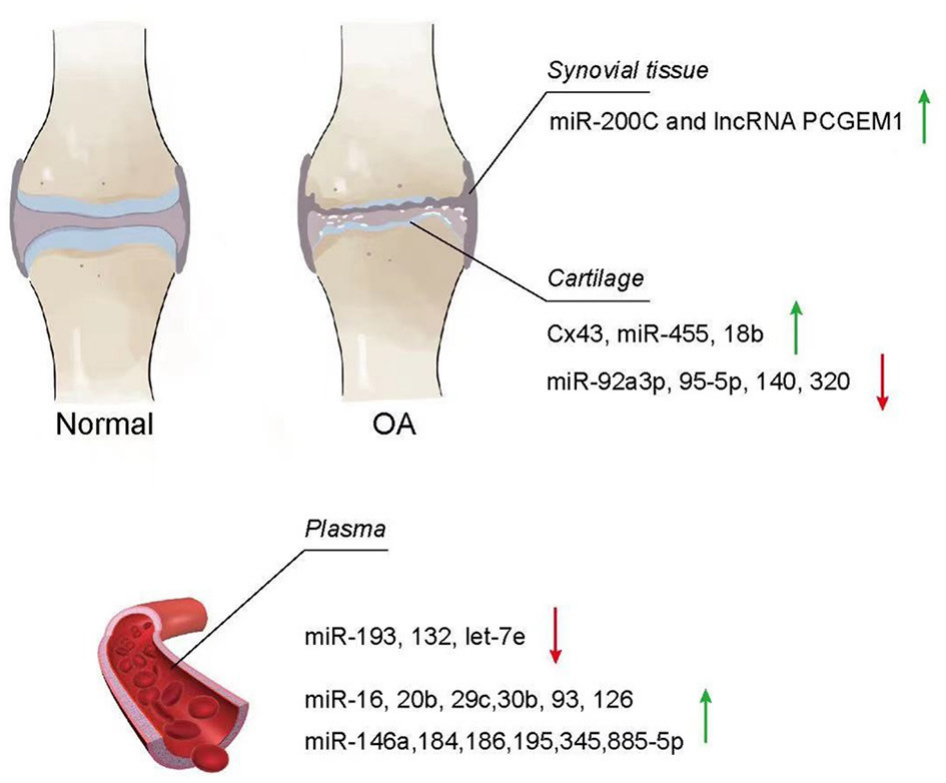
Change of EVs from osteoarthritis **(**OA). Exosomes in the synovial tissue, cartilage, and plasma of patients with OA showed different increasing and decreasing trends compared with normal patients.

A few studies have confirmed that EVs from patients with an osteoarthritic joint were significantly different from normal patients (Barile and Vassalli, 2017; Zhang et al., 2017; Zhou Q. et al., 2020) (Figure 3). For instance, significantly elevated concentrations of LncRNA PCGEM1 were detected in synovial fluid from patients with OA (Zhao and Xu, 2018). In another example, the plasma collected from patients with OA had the downregulated expressions of miR-193b, miR-132, and let-7e (Murata et al., 2010; Beyer et al., 2015; Fangang et al., 2018), while the expressions of miR-16, miR-20b, miR-29c, miR-30b, miR-93, miR-126, miR-146a, miR-184, miR-186, miR-195, miR-345, and miR-885-5p were significantly upregulated (Borgonio Cuadra et al., 2014). In addition, these EVs have a negative impact on joints, such as the propagation of inflammation and degeneration of cartilage. Based on this, it is reasonable to make the assumption that the changes in EVs from different tissues may reflect the pathophysiological conditions of the joint in some aspects and potentially serve as a diagnostic indicator of the joint disease.

#### Synovial Tissue and Synovial Fluid

Synovial fluid works as a lubricant to reduce friction with the joint and absorb shock during the movement (Choi et al., 2019). Meanwhile, it also plays an important role for nutrient and waste transportation for chondrocytes (Valachová and Oltés, 2021). Currently, researchers have investigated the contents of EVs distributed in synovial fluid and found that EVs from OA synovial fluid showed a negative impact on the articular chondrocytes, inducing the promotion of macrophage proliferation and osteoclast formation as well as activating inflammatory cells (Song et al., 2016; Kolhe et al., 2017; Rossana et al., 2017). Incremental cargoes of EVs, such as miR-200C and LncRNA PCGEM1, from the synovial tissue and synovial fluid were identified in patients with joint diseases. By comparing synovial fluid exosomes from patients with OA with those from normal patients using nanoparticle-tracking analysis (NTA) to quantify the size and concentration of exosomes within the synovial fluid and anti-CD63 antibody, as well as the specificity of these isolated particles, Withrow et al. found that miR-200C increased 2.5-fold in the synovial fluid (Withrow et al., 2016). In fact, miR-200C has been reported to contribute to IL-6-mediated inflammation (Rokavec et al., 2012). In another study, Zhao and Xu (2018) used UC to isolate exosomes from blood and synovial fluid, followed by extraction and measurement of LncRNAs in exosomes using the RNeasy kit and quantitative real-time PCR (qPCR), respectively. The results showed that LncRNA PCGEM1 [a sponge IncRNA targeting miR-770 and motivating the propagation of osteoarthritic synoviocytes (Kang et al., 2016)] expression in synovial fluid was significantly higher in early primary OA (KL scale 0–2, 2:16:2) and late primary OA (KL scale 1–4, 1:3:15:3) compared to controls (KL scale 0–1, 18:2), which in turn was higher in late OA than in early OA. Because the expression of LncRNA PCGEM1 is related to the stage of OA and is easy to isolate from synovial fluid, LncRNA PCGEM1 would be a potential diagnostic indicator.

#### Chondrocytes and Cartilage

The fundamental mechanism of OA development is the change of healthy chondrocytes to OA chondrocytes, where OA chondrocytes lose their capacity to secrete and maintain ECM, resulting in the loss of the cartilage tissue. During the transformation from chondrocytes to OA chondrocytes, the contents of EVs also change, which have been reported in recent studies. For example, Mao et al. found that miR-92a-3p was significantly downregulated in the chondrocytes of a collagenase-induced OA (CIOA) model compared with the normal cartilage using UC, which isolated exosomes, and a Total Exosome RNA & Protein Isolation Kit (Invitrogen, Carlsbad, CA, USA), which extracted RNA and proteins from exosomes. Given that miR-92a-3p directly aims at WNT5A (Mao et al., 2018b), which plays an important role in both chondrogenic differentiation and cartilage degradation (Hosseini-Farahabadi et al., 2013), and miR-92a-3p enhances H3 acetylation on aggrecan (ACAN), cartilage oligomeric matrix protein (COMP), and COL2A1 promotion, and also promoted relative cartilage matrix expression (Mao et al., 2017), and the downregulated expression of miR-92a-3p potentially be a direct indication of the development of OA. In addition, Mao et al. also found that miR-95-5p was downregulated in degraded OA chondrocyte with UC-isolated exosome, and miRNA microarray analyzed the miRNA profiles, resulting in the inhibition of histone deacetylase (HDAC) 2/8 and further leading to the downregulation of expression of cartilage-specific genes as well the obstruction of cartilage development. This result indicates that miR-95-5p could accommodate cartilage development and homoeostasis (Mao et al., 2018a). In another study, miRNA microarrays and quantitative PCR were performed in RNA from *in vitro* cartilage, and the amount of miR140 associated with chondrocyte differentiation (Miyaki et al., 2009), and miR-320 constraining MMP-13 and producing the IL-1β-stimulated catabolic effect (Meng et al., 2016) were diminished in degenerative OA chondrocytes (Swingler et al., 2012).

Interestingly, some cargoes of EVs show an increasing trend in OA chondrocytes. Varela-Eirín et al. found that the level of exosome channel protein connexin43 (Cx43) evaluated by western blot, immunofluorescence, and flow cytometry was significantly higher in EVs isolated from primary OA chondrocytes by UC. The overexpression of Cx43 in chondrocytes shows an increasing senescence effect (Varela-Eirín et al., 2019). Moreover, miR-455 was found with an increased expression in the degraded OA cartilage using a microarray, which measured the expression of miRNAs in the ATDC5 cell model of chondrogenesis and quantitative reverse transcription–PCR (qRT-PCR), which verified miRNAs. This overexpression contributes to cartilage destruction and exacerbates OA progression during the aging process (Swingler et al., 2012). MiR-181b, a negative regulator of cartilage development, was also significantly overexpressed in the cartilage of OA induced by the destabilization of the medial meniscus (DMM) by real-time PCR (Song et al., 2013). Song found that the use of a mimic or an inhibitor to alter miR-181b levels in chondroblasts and articular chondrocytes showed that the attenuation of miR-181b reduced MMP-13 expression while inducing COL-II expression.

#### Plasma

There were a number of cargoes in plasma EVs whose level was significantly lower than normal controls. Fang Gang et al. identified RNA extracted from exosomes using the miRNeasy Serum/Plasma Kit (Qiagen, Hilden, Germany) by Nanoparticletracking analysis (NTA) and transmission electron microscopy (TEM) and found that miR-193b levels were lower in OA patients than in healthy subjects. They further demonstrated that miR-193b could regulate chondrogenesis and metabolism by inhibiting HDAC3, which played a pivotal role in suppressing the expression of specific genes in human chondrocytes (Fangang et al., 2018). Furthermore, Murata observed that plasma miR-132 from patients with OA was significantly lower by qRT-PCR. The receiver operating characteristic (ROC) analysis showed that miR-132 test at a cutoff value of 67.1 pmol/L was detected in individuals with OA at 84.0% of sensitivity and 81.2% of specificity (Murata et al., 2010). In another study, Beyer confirmed that the decreased levels of plasma let-7e from patients with OA were associated with the progression of hip/knee OA after microarray screen and validated 12 miRNAs by real-time PCR, which required total hip or knee arthroplasty (Beyer et al., 2015). While the level of some miRNAs was increased in plasma EVs. In the plasma from patients with primary knee OA, Borgonio identified 12 overexpressed miRNAs (miR-16, miR-20b, miR-29c, miR-30b, miR-93, miR-126, miR-146a, miR-184, miR-186, miR-195, miR-345, and miR-885-5p) by TaqMan profiling Low Density Array (TLDA), which screened 380 miRNAs, and qRT-PCR singleplex, which validate miRNA array results (Borgonio Cuadra et al., 2014) related with the development of the disease.

Taken together, the changes in EV quantity and composition reflect the progression of OA, particularly, the examination of miR-200C and LncRNA PCGEM1 in synovial fluid, as well as the monitoring of miR-132 and let-7e in plasma. It contributes to the diagnosis of OA when miR-200C increases 2.5-fold in synovial fluid and miR-132 markedly decreases in plasma. In addition, a remarkable rise of LncRNA PCGEM1 usually indicates the advanced stage of OA. Furthermore, patients with extremely declined plasma let-7e should be considered for the surgical treatment of total hip or knee arthroplasty. Overall, EVs do have the potential of diagnostic biomarkers for OA; however, the feasibility and value of EVs remain to be further investigated. Noteworthy, although EVs may also reflect the early development of ACD, limited studies have been conducted in this aspect. More importantly, if the early cartilage damage can be screened by EV changes and treated appropriately, it may be possible to prevent its eventual development into OA. Although the studies on the crosstalk between EV changes and ACD/OA are still at the beginning stage, the potential of EVs for the early diagnosis and classification of joint diseases is very promising and should become a focus of future research.

## Role of EVs in the Treatment of Articular Cartilage Lesions and Degenerative Joint Diseases

Osteoarthritis is primarily caused by post-traumatic cartilage damage or age-related cartilage degeneration, involving synovium, subchondral bone, and periarticular tissues, to result in pain, joint stiffness, a limited range of motion (ROM), and other symptoms, which seriously affect the life quality of patients (Loeser et al., 2012). Aging, obesity, gender, and genetic predisposition are the critical risk factors leading to the complex pathogenesis of OA (Johnson and Hunter, 2014). At present, it is believed that the pathological process of OA development is the result of multiple factors such as mechanical stimulation, an intra-articular inflammatory reaction, the disruption of cartilage homeostasis, and the changes in subchondral bone microstructure (Hashimoto et al., 2008; Goldring and Marcu, 2009). Chronic mechanical stresses (such as joint overuse) lead to cartilage injury, and the degradation products of an injured cartilage can stimulate synovium and trigger an inflammatory response (Mathiessen and Conaghan, 2017). In addition, inflammatory synovium produces pro-inflammatory factors, upregulating the expression of matrix degrading proteinases (Kim et al., 2006; de Lange-Brokaar et al., 2012). Meanwhile, oxidative stress related to inflammation or senescence causes mitochondrial dysfunction and chondrocytes apoptosis (McCulloch et al., 2017; Tofino-Vian et al., 2017). The loss of anabolic/catabolic equilibrium destroys cartilage homeostasis, resulting in the reduction of chondrocytes, loss of ECM, and eventual cartilage degeneration (Mueller and Tuan, 2011). Furthermore, the imbalance between bone resorption and formation in bone remodeling leads to subchondral bone sclerosis and osteophyte formation (Sellam and Berenbaum, 2010). These osteochondral changes will aggravate synovitis, which in turn leads to a vicious cycle that further deteriorates the cartilage tissue (Goldring and Goldring, 2006).

To date, to slow or prevent the progression of OA, no effective clinical solution exists. Conventional treatments often involve the use of drugs (such as non-steroidal anti-inflammatory drugs and NSAIDs) to alleviate pain symptoms, which indirectly cause excessively unaware movement of the affected joints by patients, further aggravating the condition. In addition, these drugs can potentially cause severe side effects such as gastrointestinal complications, myocardial infarction, and stroke (Silverstein et al., 2000; Kearney et al., 2006; Antman et al., 2007). Arthroplasty, which is thought to be only applicable to the elderly patients in the terminal stage (Zhang et al., 2008), has disadvantages, such as high cost, large wound surface, and limited life span of the implants, limiting its utilization in an increasing number of young patients. Other surgical methods, such as microfracture and autologous chondrocyte implantation (ACI), fail to avoid the lesions of the donor site and lead to the formation of fibrocartilage, resulting in an unsatisfied clinical outcome (Jiang et al., 2011). In recent years, stem cell therapies, especially the transplantation of MSCs, have been widely used in the field of regenerative medicine. In spite of achieving some remarkable results in ACD and early OA treatment (Murphy et al., 2003; Centeno et al., 2008; Jo et al., 2017) many safety risks limit its wide application in clinical practice, such as immune rejection (Zhang et al., 2013) and tumorigenic potential of transplanted cells (Nori et al., 2015). With the deepening of research, an increased amount of evidence showed that the beneficial effects of stem cell transplantation might not be limited to its multi-differentiation potential but more related to the paracrine secretion of trophic factors, among which EVs are considered to be one of the most critical functional components (Meirelles et al., 2009; Jin et al., 2011; Wu et al., 2011). Based on this, there are more and more studies on the role of different EV types in joint diseases, and EVs-based acellular therapy is becoming a popular research topic. Exosomes and MVs, in particular, have been demonstrated their therapeutic potential for OA treatment and cartilage repair.

### Exosomes

**Figure 4:**
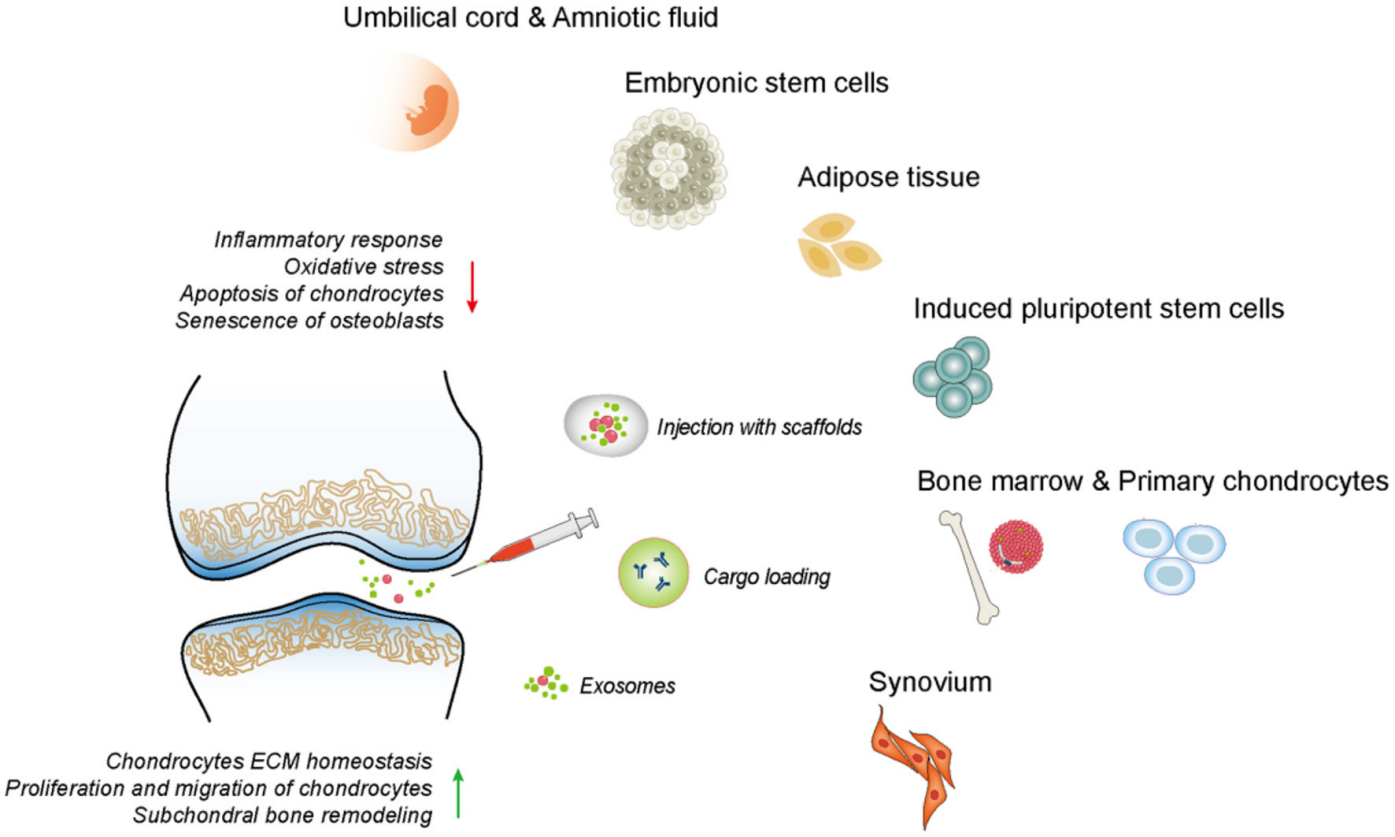
The therapeutic effects of exosomes derived from different tissues on OA. The exosomes could mediate intercellular communication and regulate diverse cell phenotype including inflammatory response, ECM synthesis, chondrocytes proliferation, migration, apoptosis, etc.

After being released by parent cells, exosomes can directly activate/inhibit target cells by binding to relevant receptors or transfer and translate the genetic material into functional proteins by endocytosis, which mediates intercellular communication and gene regulation, thereby improving the cellular function (Boyiadzis and Whiteside, 2015). As a signaling molecule involved in cellular interaction, exosomes can either accelerate the progression of OA or the promotion of articular cartilage repair (Zhou Q. F. et al., 2020). Specifically, the exosomes secreted by therapeutic cells, especially MSCs, have been used for the treatment of OA by transferring their bioactive substances to regulate the damaged tissue environment and coordinate the subsequent regenerative process (Figure 4). The mechanisms of exosomes for cartilage repair include the mechanisms for regulating immune response, inhibiting chondrocytes apoptosis, and matrix degradation, promoting the proliferation and chondrogenesis of *in situ* stem cells, as well as the directional migration to an injury site. In addition, gene modification and tissue engineering can be used to modify the exosomal secretion and contents, to obtain more ideal therapeutic exosomes to improve their therapeutic efficacy.

#### Mesenchymal Stem Cells

Because MSCs secrete more exosomes than other cells, MSCs always serve as the donor cells of the preferred exosomes (Yeo et al., 2013). MSC-derived exosomes can be easily extracted from many tissues, including but not limited to embryo, adipose tissue, bone marrow, synovium, umbilical cells, and amniotic fluid. The role of MSCs-Exos in the treatment of ACD and OA varies due to the difference of their MSC origin.

##### Exosomes From Human Embryonic Stem Cell-Induced MSCs

Embryonic stem cells are pluripotent stem cells isolated from early embryos or primitive gonads, which can differentiate into different functional cells, with the characteristics of unlimited proliferative and self-renewal potential (Biswas and Hutchins, 2007). Zhang et al. (**Figure 6A**) discovered the repair effect of exosomes from human embryonic stem cell-induced MSCs (hESC-MSCs-Exos) on the cartilage tissue for the first time. In the rat model of a knee cartilage defect, the appearance and histological scores of a knee joint were significantly improved in the exosome-treated group (Zhang et al., 2016). At 12 weeks, the cartilage and subchondral bone defects were completely repaired, the hyaline cartilage was well integrated with the surrounding cartilage, and the ECM deposition was close to normal while the control group only showed a fibrous tissue repair. In another rat model of temporomandibular joint OA (TMJ-OA), hESC-MSCs-Exos were shown to promote the synthesis of IL-1β-inhibited s-GAG and to suppress the production of IL-1β-induced MMP-13 and NO, resulting in the promotion of cellular proliferation and directional migration, which further restored matrix homeostasis and alleviated inflammatory response (Zhang et al., 2019). In further studies, the authors attributed this combined remediation to the exosomal CD73-mediated adenosine activation of ERK/AKT signaling pathway and the immunomodulation of inducing the transformation of macrophages from M1 pro-inflammatory to M2 anti-inflammatory phenotype (Zhang et al., 2018). Wang et al. in their study found that hESC-MSCs-Exos delayed the progress of OA by balancing the synthesis and degradation of ECM (Wang et al., 2017). In the mice model of knee OA obtained by DMM, hESC-MSCs-Exos increased the synthesis of COL-II and suppressed the expression of ADAMTS-5, resulting in restoring matrix homeostasis and maintaining chondrocyte phenotype.

##### Exosomes From Adipose Tissue-Derived MSCs

Adipose tissue-derived MSCs (AD-MSCs) are stem cells isolated from adipose tissues, which have shown good efficacy in the treatment of OA due to their abundant tissue sources and good tolerance in an ischemic cartilage microenvironment (Lindroos et al., 2011). Tofino-Vian et al. found that the exosomes from AD-MSCs (AD-MSCs-Exos) prevented the senescence of OA osteoblasts by suppressing inflammation and oxidative stress (Tofino-Vian et al., 2017). In an *in vitro* inflammatory model, AD-MSCs-Exos inhibited the activity of β-galactosidase, reduced the production of IL-6 and prostaglandin E_2_ (PGE_2_), and significantly downregulated the DNA damage caused by the potential alterations and oxidative stress in mitochondrial membranes. In addition, exosomes redressed the abnormal osteoblastic metabolism and regulated the bone remodeling during joint degeneration, suggesting that they might treat OA through an anti-senescence effect. The authors further found that AD-MSCs-Exos might reduce the DNA binding affinity of transcription factor c-jun activating protein-1 (AP-1) and nuclear factor-κB (NF-κB), and then downregulated the transcription of MMPs, minimizing the expression of inflammatory and catabolic mediators (Tofino-Vian et al., 2018). Woo et al. also evaluated the therapeutic potential of human AD-MSCs-Exos in OA treatment through the monosodium iodoacetate (MIA) rat and DMM mouse model (Woo et al., 2020). The results indicated that the isolated exosomes not only facilitated the proliferation and migration of chondrocytes and maintained the homeostasis of chondrocyte matrix, but also suppressed the infiltration of M1 macrophages into synovium, hence preventing cartilage degeneration and delaying the progression of OA. Moreover, it was reported that AD-MSCs-Exos could enhance the proliferation and chondrogenic potential of periosteal cells by upregulating miR-145 and miR-221, respectively, and protect chondrocytes from H_2_O_2_-induced apoptosis (Zhao et al., 2020). In another study, Wu et al. found that the exosomes from infrapatellar fat pad MSCs (MSC^IPFP^-Exos) could suppress matrix degradation, promote matrix synthesis and reverse IL-1β-induced apoptosis, which ameliorated the pathological severity of OA (Wu et al., 2019). They attributed this chondroprotective function to the miR-100-5p-regulated inhibition of an mTOR-autophagy pathway. MiR-100-5p could bind to 3′-untranslated region (3′-UTR) of mTOR to reduce its expression, which activated the protective autophagy of chondrocytes, restoring microenvironmental homeostasis of the cartilage.

##### Exosomes From Bone Marrow MSCs

Bone marrow MSCs (BM-MSCs) are the earliest stem cells discovered in humans and have been widely used in experimental research and clinical applications. They are considered to be the most ideal seed cells for tissue engineering because of easy isolation process, enhanced proliferation capacity, and excellent differentiation potential (Strioga et al., 2012). Cosenza et al. (**Figure 6B**) found that the exosomes from BM-MSCs (BM-MSCs-Exos) could inhibit the expression of catabolic markers (MMP-13, ADAMTS-5) and inflammatory markers [inducible NO synthase (iNOS)], restore the production of chondrocyte markers (COL-II, ACAN), and suppress the apoptosis of chondrocytes and activation of macrophages in a while, resulting in the reduction of inflammatory level, the promotion of cartilage repair, and the alleviation of OA (Cosenza et al., 2017). This suggested that BM-MSCs-Exos might have the anti-inflammatory and anti-apoptotic function. Similar conclusions were drawn from a series of experiments. Qi et al. reported that the absorption of BM-MSCs-Exos by chondrocytes reversed the decrease of chondrocytes viability and mitochondrial-induced apoptosis in response to IL-1β, which was associated with the inhibition of phosphorylation in p38 and ERK, and the promotion of phosphorylation in AKT (Qi et al., 2019). Other studies demonstrated that exosomal miR-9-5p (Jin et al., 2020a) and miR-127-3p (Dong et al., 2021) secreted by BM-MSCs could alleviate the inflammatory response and OA-like damage *via* targeting syndecan-1 (SDC1) and cadherin-11 (CDH11), respectively. To further understand the molecular mechanism of this anti-inflammatory effect, Vonk et al. examined the subcellular localization of p56 (a subunit of NF-κB) and found that BM-MSCs-Exos blocked the activation of an NF-κB pathway by restraining the phosphorylation of IκBα (Vonk et al., 2018). Moreover, these exosomes could also promote chondrogenesis and inhibit the hypertrophic differentiation of chondrocytes by upregulating the expression of SRY-box transcription factor 9 (SOX9) and WNT7A, and downregulating the expression of RUNX2, COL10A1, and alkaline phosphatase (ALP).

**Figure 5:**
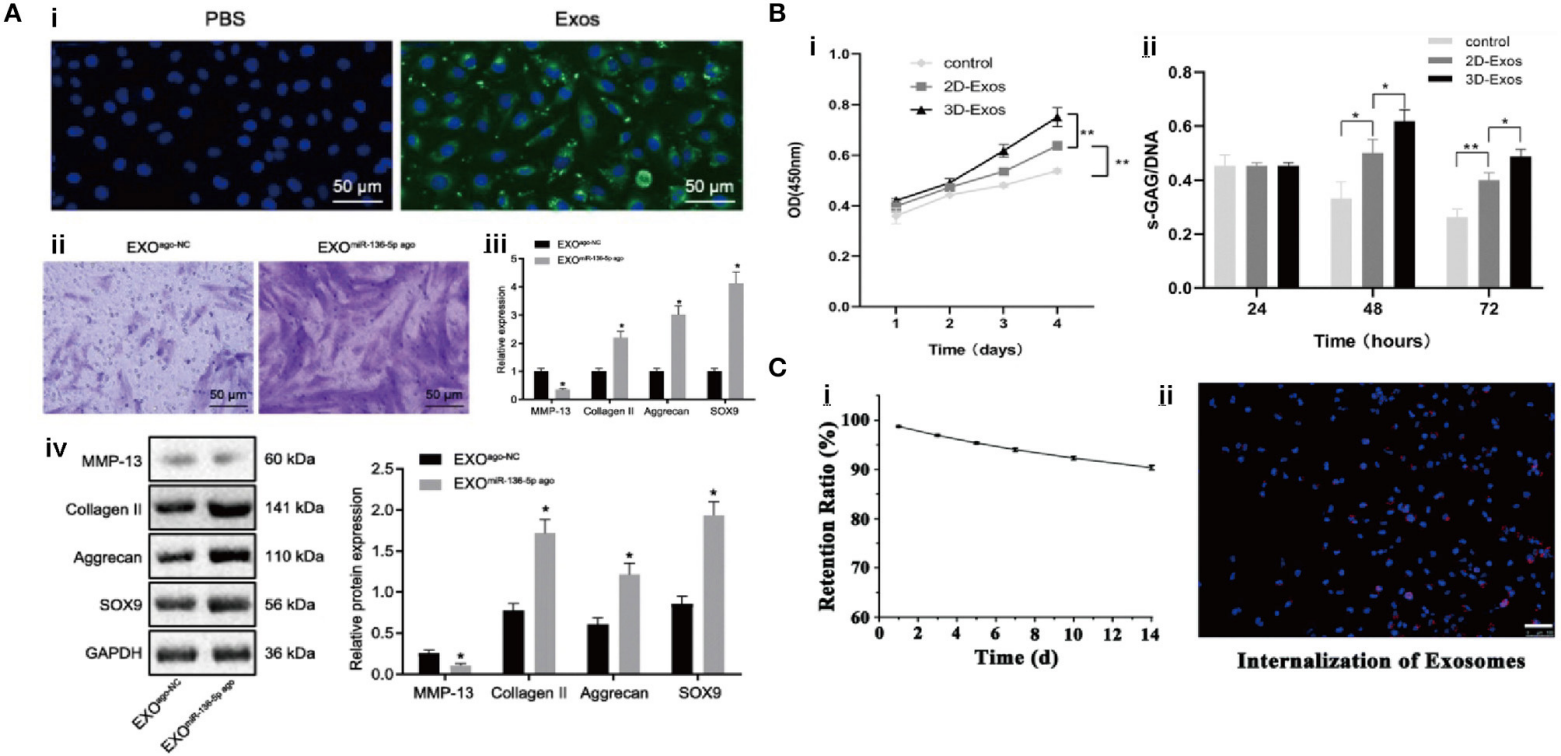
The interaction between exosomes and chondrocytes. **(A)** Exosomes can promote chondrocyte migration and chondrogenic gene expression (Chen et al., 2020). **(B)** Exosomes can upregulate cellular growth and GAG secretion (Yan and Wu, 2020). **(C)** Exosome retention rate with hydrogel and cell uptake (Viñuela-Berni et al., 2015).

In the study of Chen (Figure 5A), it was demonstrated that exosomal miR-136-5p secreted by BM-MSCs facilitated chondrocyte migration *in vitro* and suppressed cartilage degeneration *in vivo* (Chen et al., 2020). He et al. reported that BM-MSCs-Exos alleviated the inhibitory effect of IL-1β on the proliferation and migration of chondrocytes and redressed the imbalance of matrix anabolism *in vitro*, and downregulated calcitonin gene-related peptide (CGRP) and iNOS in the dorsal root ganglion (DRG) tissue of sodium iodoacetate-induced rat model, consequently reducing the pain response (He et al., 2020a). In another experiment, Zhou et al. acquired a special kind of exosomes from polydactyly bone marrow-derived MSCs (pBMSCs-Exos), which showed an improved therapeutic effect compared to BM-MSCs-Exos by regulating chondrogenesis *via* a BMP4 signal pathway in an OA mouse model (Zhou X. et al., 2020). Collectively, these results suggested that BM-MSCs-Exos might treat OA by promoting the proliferation and migration of chondrocytes.

In addition, BM-MSCs-Exos could exert a beneficial therapeutic effect on OA by improving cartilage breakdown and subchondral bone remodeling (Zhu et al., 2020). Li et al. found that the application of BM-MSCs-Exos in the lumbar facet joint OA (LFJ-OA) mice model could reduce the expression of CGRP, inhibit the sensory nerve invasion and angiogenesis of subchondral bone, hence relieving chronic pain (Li J. et al., 2018). Besides, these exosomes could reduce cartilage degeneration, suppress the expression of tartrate-resistant acid phosphatase (TRAP) and the activation of RANKL–RANK-tumor necrosis factor receptor-associated factor 6 (TRAF6) signaling pathway to promote subchondral bone remodeling.

##### Exosomes From Synovial Membrane-Derived MSC

Synovial membrane-derived MSCs (SMMSCs) can be obtained by arthroscopy, and the synovial tissue is abundant in nature and possesses regenerative capability. Because synoviocytes and chondrocytes originate from the same cell population, SMMSCs possess characteristics similar to chondrocytes, which make them an attractive cell source for cartilage repair (Sakaguchi et al., 2005). Qiu et al. found that exosomal miR-129-5p from human SMMSCs targeted the 3′-UTR of high mobility group protein-1 (HMGB1), which suppressed the inflammatory response and apoptosis of chondrocytes mediated by IL-1β, alleviating the severity of OA (Qiu et al., 2021).

Even though the exosomes from SMMSCs (SMMSCs-Exos) hold the potential for cartilage repair whose effectiveness was inadequate when compared to other cell sources. For example, Zhu et al. compared the efficacy of SMMSCs-Exos and exosomes from the induced pluripotent stem cell-derived MSCs (iMSCs-Exos) in the treatment of OA in a CIOA mouse model (Zhu et al., 2017). The results showed that both of them could delay the progression of OA by enhancing the proliferation and migration of chondrocytes, but the effect of iMSCs-Exos was stronger. In the SMMSCs-Exos-treated group, the cartilage injury was not completely repaired, and the irregular scars were observed on the cartilage surface. While the repaired cartilage in the iMSCs-Exos-treated group showed typical hyaline features, and the surface of cartilage was smooth and regular similar to normal cartilage.

##### Exosomes From Umbilical Cord MSCs

Umbilical cord MSCs (*UC-MSCs*) are a primitive type of stem cells isolated from an amniotic membrane, a cord lining, Wharton's jelly, and a perivascular region of which phenotype is between adult stem cells and embryonic stem cells. They not only have the common characteristics of MSCs but also have faster self-renewal properties. Compared to *BM-MSCs* and *ESC-MSCs, UC-MSCs* have the advantages of painless collection procedure and non-ethical controversy (Ding et al., 2015). Jiang et al. reported the regulatory function of the exosomes from human umbilical cord Wharton's jelly MSCs (hWJMSC-Exos) in a joint microenvironment *via* a rat model of cartilage defect, and further attributed this function to the inhibition of inflammatory response by promoting the polarization of macrophages toward M2 phenotype (Jiang et al., 2021). In addition, Yan et al. (Figure 5B) found that the exosomes from UC MSCs (UC-MSCs-Exos) could promote chondrocyte proliferation, migration, and matrix synthesis; and inhibit apoptosis, which was correlated to the activation of TGF-β1 and Smad2/3 signaling (Yan and Wu, 2020). Hu et al. carried out a similar experiment, in which miR-23a-3p-abundant-UC-MSCs-Exos promoted cartilage regeneration, and further demonstrated that exosomal miR-23a-3p upregulated AKT expression through targeting phosphatase and tension homolog deleted on chromosome 10 (PTEN), which activated PTEN/AKT signaling, accelerated the proliferation, differentiation, and the migration of chondrocytes and BMSCs (Hu et al., 2020).

##### Exosomes From Amniotic Fluid Stem Cells

In recent years, amniotic fluid stem cells (AFSCs) are a kind of non-tumorigenic pluripotent cells, which have been proved to have an enhanced immunomodulatory function and non-tumorigenic properties. It is considered that AFSCs are a valuable source of cell therapies for degenerative diseases due to their easy acquisition and few ethical concerns (Zhou J. et al., 2014). Zavatti et al. in their experiments demonstrated the chondroprotective function of hAFSC-Exos for the first time. They compared the efficacy of the exosomes from AFSCs (AFSC-Exos) with their cell source in an MIA-induced OA rat model, and the results revealed that the hyaline cartilage formation with good surface regularity was observed in the exosome-treated group after 3 weeks, and the pain tolerance level and histological score of the exosome-treated group were superior to that of the AFSC-treated group. In addition, these exosomes alleviated the inflammatory response by inhibiting M1 polarization (Zavatti et al., 2020).

In summary, MSCs-Exos therapy is a promising nanotechnology for cartilage repair. Although it has been reported that AD-MSCs-Exos showed an improved therapeutic effect compared to BM-MSCs-Exos and SMMSCs-Exos (Li et al., 2021), more comparative experiments are required to conclusively determine which MSCs can be considered as the best choice for the cell source to harvest exosomes.

### Adult Cell

**Figure 6:**
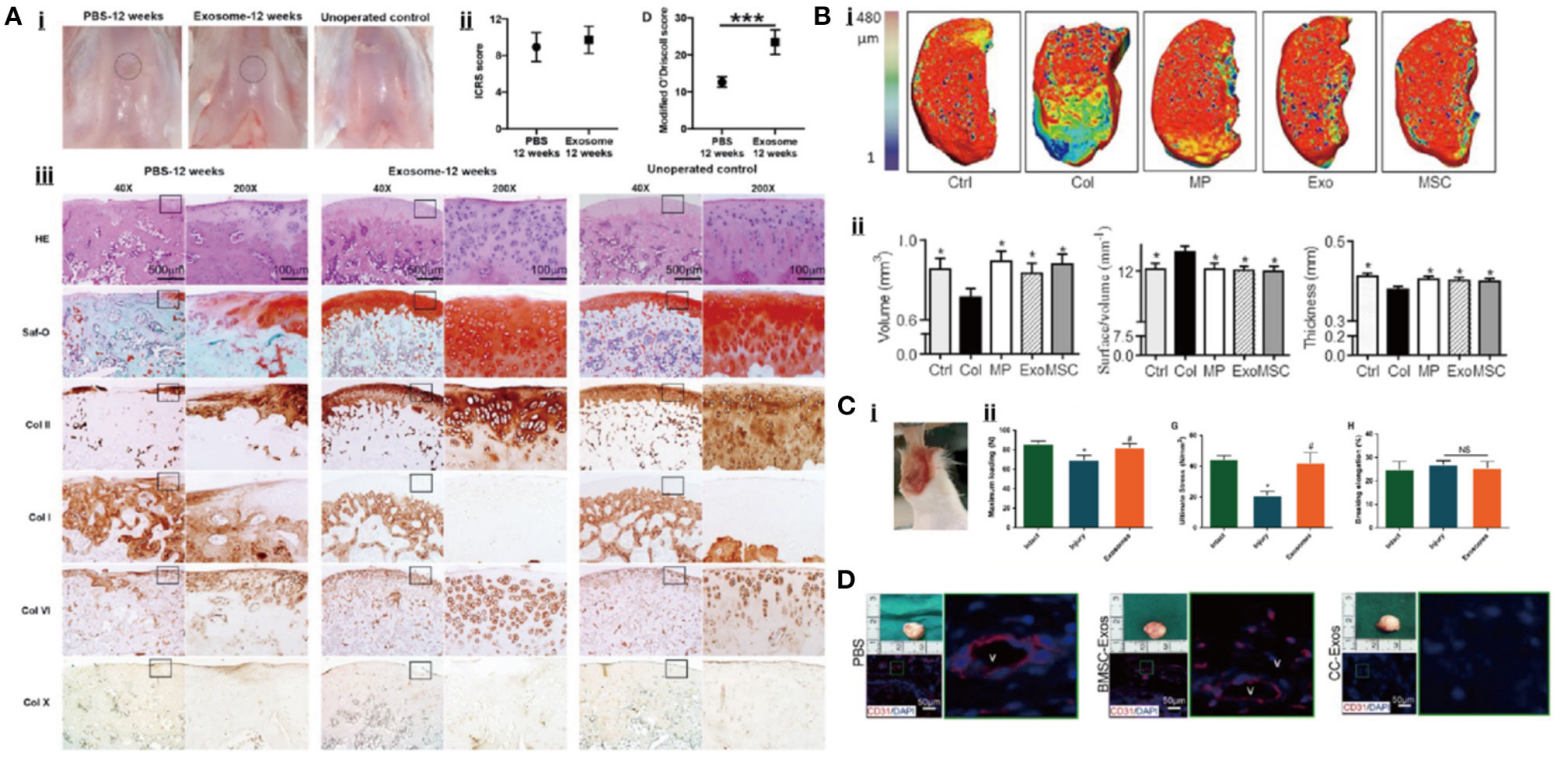
Exosomal therapeutic effect in cartilage repair. **(A)** Cartilage tissue regeneration in rat after the treatment (Zhang et al., 2016). **(B)** Exosomes can regenerate the cartilage tissue in a mice OA model (Cosenza et al., 2017). **(C)** The therapeutic effect of exosomes for tendon repair (Wang et al., 2019). **(D)** Exosome promotes the vascular formation within the bone (Chen et al., 2018).

Apart from MSCs, some studies suggest that the exosomes secreted by adult cells from different tissues of the joint may be involved in the regulation of joint homeostasis as well. In the study of Chen (Figure 6D), chondrocytes-derived exosomes (CC-Exos) were identified to play a role in guiding the ectopic chondrogenesis of CPCs with enhanced ability to promote hyaline cartilage formation compared to BM-MSCs-Exos (Chen et al., 2018). In their study, CC-Exos promote COL-II deposition and reduce angiogenesis. More importantly, the phenotype of new cartilage was stable. However, the generated cartilage in the BM-MSCs-Exos-treatment group, as a positive control, was characterized by hypertrophy with vascular ingrowth. Zheng et al. also found that the exosomes from primary chondrocytes cultured in normal environment (D0-Exos) could restore mitochondrial dysfunction and polarize macrophages to M2 phenotype, which effectively prevented the progression of OA (Zheng et al., 2019). In addition, exosomal miR-8485 from chondrocytes has been found to target Dapper1 Antagonist of Catenin-1 (DACT1) to induce p-GSK-3β (Ser9), which activated Wnt/β-catenin pathways, resulting in the chondrogenic differentiation of BMSCs (Li et al., 2020).

Wang et al. (Figure 6C) proved for the first time that the exosomes from tendon stem cells (TSCs-Exos) could alleviate the pathological alterations of tendon (Wang et al., 2019). In the rat model of achilles tendon tendinopathy established by collagenase-I injection, TSCs-Exos increased the expression of an tissue inhibitor of metalloproteinase-3 (TIMP-3) and COL-1a1 as well as the ultimate stress and maximum loading of the model, balancing the tendon microenvironment and improving the biomechanical properties of the damaged tendon. Xu et al. found that the exosomes from tenocytes could induce the tenogenic differentiation of MSCs in a TGF-β-dependent manner, which promoted tendon repair and regeneration, providing a new therapeutic strategy for tendon abnormalities-related OA (Xu et al., 2019).

#### Exosomes From Genetically Modified Cells and Engineered Exosomes

MicroRNAs are a group of non-coding RNAs, containing ~22 nucleotides, which can regulate posttranscriptional gene expression by binding to the 3′-UTR of the target mRNAs, resulting in translation inhibition or target gene degradation (Ghildiyal and Zamore, 2009). A few studies have confirmed that miRNAs are effective regulators of some key genes and signaling pathways in OA, which may play an important role in mediating MSC-exosomes against OA (Iliopoulos et al., 2008; Yang et al., 2011; Ham et al., 2012; Matsukawa et al., 2013; Ning et al., 2013; Meng et al., 2016; Kim D. et al., 2020). In this regard, some scholars have proposed that MSCs could be modified by gene modifications, such as the transfection to improve the expression level of specific genes, so as to obtain the exosomes containing target miRNAs for the treatment of OA.

Tao et al. found that SMSCs-Exos could enhance the proliferation and migration of chondrocytes through the WNT5a/b-YAP signaling pathway but also suppress the expression of SOX9, causing a decreased secretion of ECM (Tao et al., 2017). To overcome this side effect, the research team obtained miR-140-5p over-expressed SMSCs by using lentivirus transfection and demonstrated that the exosomes secreted from the modified stem cells (SMSCs-140-Exos) could effectively suppress the expression of RalA, which reversed the inhibition of SOX9, thus restoring the efficiency of ECM synthesis. In the rat model established by anterior cruciate ligament transection (ACLT), SMSCs-140-Exos showed an enhanced therapeutic effect in terms of slowing down the progression of early OA when compared to unmodified SMSCs-Exos. Wang et al. carried out a similar study (Wang et al., 2021). In their experiment, they observed that SMSC-Exos had an insufficient effect on the ECM secretion from OA chondrocytes, and this issue was subsequently corrected by overexpressing miR-155-5p in SMSCs. Further experiments demonstrated that SMSCs-155-5p-Exos specifically targeted RUNX2 to promote the secretion of ECM. Mao et al. found that the exosomes secreted by miR-92a-3p-overexpressed human MSCs (hMSCs-miR-92a-3p-Exos) could directly target WNT5A, which further upregulated the expressions of COL2A1, COL9A1, ACAN, SOX9, and COMP, and downregulated the expressions of COL10A1, RUNX2, and MMP-13, promoting chondrogenesis and inhibiting cartilage degradation *in vivo* and *in vitro* (Mao et al., 2018a). In another study, the exosomes from miR-95-5p-over-expressed primary chondrocytes (AC-miR-95-5p-Exos) directly targeted HDAC2/8 to facilitate ECM expression and an enhanced hyaline cartilage formation, hence delaying the progress of OA as well (Mao et al., 2018a). Sun et al. in their study reported the reduced expression of SOX9 and increased expression of MMP-13 in OA chondrocytes (Sun et al., 2019). This phenomenon was effectively reversed by the exosomes derived from miR-320c-over-expressed human BMSCs (hBMSC-320c-Exos), which further enhanced the chondrogenic potential of BMSCs by promoting the proliferation and migration of chondrocytes. Moreover, Jin et al. demonstrated that the exosomes secreted by miR-26a-5p-overexpressed human bone MSCs alleviated the damage of synovial fibroblasts by targeting prostaglandin-endoperoxide synthase 2 (PTGS2), thereby attenuating OA (Jin et al., 2020b). In addition, He et al. explored the protective mechanism of the exosomes from miR-210-over-expressed BMSCs (BMSCs-210-Exos) against lipopolysaccharide (LPS)-induced cartilage injury, and the results suggested that the anti-apoptotic function in chondrocytes was related to the inhibition of tumor necrosis factor receptor superfamily member 21 (Tnfrsf21) expression and the reduction of NF-κB pathway by BMSCs-210-Exos (He et al., 2020b).

In addition to miRNAs, LncRNAs is another important segment of signal exchange. LncRNAs are a group of non-coding RNAs with no protein coding potential and more than 200 nucleotides in length, which play an important regulatory role in cell proliferation, migration, and apoptosis (Tang and Hann, 2018). The mechanism of exosomal LncRNA in OA treatment was revealed by Liu et al. (2018). In the CIOA mice model, the exosomes secreted by LncRNA-KLF3-AS1-over-expressed MSCs (MSC^KLF3−AS1^-Exos) increased the expression of chondrogenic genes (ACAN and COL2A1) and decreased the expression of hypertrophy markers (RUNX2 and MMP-13), thus promoting the proliferation of chondrocytes. Moreover, the authors also found that MSC^KLF3−AS1^-Exos could inhibit the apoptosis of chondrocytes *via* miR-206/G-protein-coupled receptor kinase interacting protein-1 (GIT1) axis.

In addition to the amplification of a specific miRNAs fragment within exosomes through a genetic engineering approach, exogenous drug intervention can also regulate the exosomal secretion and contents to enhance the therapeutic effectiveness of MSCs-Exos for OA. Wang et al. found that TGF-β1 could stimulate MSCs to secrete exosomes containing miR-135b (TGF-β1-Exos), which in turn downregulated specificity protein 1 (Sp1), thereby enhancing the proliferation of chondrocytes (Wang et al., 2018). Further studies suggested that exosomal miR-135b from BMSCs targeted mitogen-activated protein kinase 6 (MAPK6), which promoted M2 polarization of synovial macrophages, facilitating the repair of OA cartilage (Wang and Xu, 2021). Similarly, Kim et al. demonstrated that IL-1β could increase the content of miR-147b in MSC-Exos, thereby significantly suppressing the expression of inflammatory cytokines and enhancing the anti-inflammatory activity of MSC-IL-Exos through the inhibition of an NF-κB pathway mediated by exosomal miR-147b (Kim et al., 2021). Moreover, it was reported that kartogenin (KGN) preconditioning could effectively avoid the depression of RUNX1 in BMSC-Exos-treated chondrocytes, hence improving the properties of BMSC-Exos and providing better cartilage repair performance (Liu et al., 2020).

Being a nano-carrier naturally secreted by cells, exosomes also have advantages, such as small size, large surface area, strong targeting, high bioavailability, and low immunogenicity, providing a new idea for the drug delivery therapy of OA (Zhou Q. F. et al., 2020). Xu et al. fused the MSC-binding peptide E7 with the exosomal membrane protein Lamp 2b to obtain the SF-MSC-targeted exosomes (E7-Exos). These exosomes effectively mediated the entry of KGN into SF-MSCs and induced a higher degree of chondrogenic differentiation of SF-MSCs than KGN alone or the exosomal KGN without E7 (Xu et al., 2021). In another study, Tao et al. loaded WIKI4, an inhibitor of tankyrase (tankyrase is a target locus for treating OA), into CC-Exos (WIKI4-cExos) to achieve the targeted delivery of WIKI4 to the cartilage, which not only delayed the progression of OA but also reduced the side effect that WIKI4 could cause bone loss in addition to cartilage protection (Tao and Guo, 2020).

### Microvesicles

Unfortunately, limited literature studies are conducted with a focus on the application of MVs in the joint disease, and being a key component of EVs-based acellular therapy, MVs should not be overlooked. Although MVs and exosomes overlap in size and content, they contain different proteins and RNAs, suggesting that they may mediate OA treatment or cartilage repair through different molecular mechanisms.

Cosenza et al. were the first one to compare the efficacy of MVs and exosomes in the treatment of OA and showed no significant difference in terms of anti-inflammatory and chondroprotective functions both *in vitro* and *in vivo* (Cosenza et al., 2017), while Tofino-Vian et al. showed a trend for MVs to exert a better anti-inflammatory effect than exosomes and attributed this to the overexpression of anti-inflammatory protein annexin A1 (AnxA1) (Tofino-Vian et al., 2018). This finding was consistent with Headland et al. who reported that neutrophil-derived MVs are enriched in AnxA1, which activates the expression of anabolic genes (COL2A1, SOX9) in chondrocytes, decreases the release of IL-8 and PGE_2_, and increases the production of TGF-β by interacting with its receptor formyl peptide receptor 2 (FRP2), thereby inducing the deposition of ECM (Headland et al., 2015).

In another *in vitro* assay, Tofino-Vian et al. found that AD-MSC-MVs reduced oxidative stress in chondrocytes (Tofiño-Vian et al., 2018). Given oxidative stress disrupting normal physiological signaling and inducing inflammatory changes, cartilage degradation, and OA progression, this particular finding is encouraging. The presence of MVs reduced the accumulation of 4-hydroxy-2-nonenal- (HNE-) modified proteins and increased the expression of peroxiredoxin 6 (an antioxidant enzyme) in OA chondrocytes stimulated by IL-1β, suggesting a protective effect of MVs against oxidative stress. In addition, MVs demonstrated their ability to inhibit IL-1β-induced apoptosis in chondrocytes and to promote chondrocyte proliferation by entering cartilage in a CD44-dependent manner (Xiang et al., 2017).

In addition to its direct anti-inflammatory and chondroprotective function, MVs also have a cell delivery role in promoting the targeted migration and adhesion of MSCs to the damaged cartilage surface. *In vitro* tests have confirmed that MVs could play an active role in intra-articular MSC homing by increasing the expression of GPIIb/IIIa, CXCR4, ITGβ1, and ITGα2, thereby promoting cartilage regeneration (Liang et al., 2020).

Taken together, MVs as a new therapeutic tool possess the potential for cartilage repair. Further studies should be conducted to comprehensively evaluate the therapeutic effect in animal models and to identify the effective dosage for downstream clinical translation.

## Discussion

Due to gradual aging and obesity of society, OA has become the most common degenerative joint disease in human society, and it is very important to find new scientific and effective therapies in view of the shortcomings of existing treatments. A large number of preclinical studies have demonstrated that EVs could alleviate the progression of OA by repairing and regenerating the damaged cartilage (Table 3), which has led to an increasing interest in the diagnostic and therapeutic value of EVs in joint diseases.

**Table 3:** The therapeutic effects and underlying mechanisms of exosomes on articular cartilage defect/osteoarthritis (ACD/OA).

**Exosomes**	**Biological effects**	**Mechanisms of actions**	**References**
*hESC-MSCs-Exos*	1.Complete restoration of cartilage and subchondral bone	1.Adenosine activation of AKT, ERK, and AMPK signaling	Zhang et al., 2016
	2.Reduce inflammation, improve subchondral bone architecture	2.Enhance s-GAG synthesis, suppress IL-1β-induced NO and MMP-13 production	Zhang et al., 2019
	3.Alleviate cartilage destruction and matrix degradation	3.Increase COL-II synthesis and decrease ADAMTS-5 expression	Wang et al., 2017
*AD-MSCs-Exos*	1.Correct abnormal osteoblast metabolism	1.Downregulate senescence-associated β-galactosidase activity and the accumulation of γH2AX foci	Tofino-Vian et al., 2017
	2.Prevent the senescence of OA osteoblasts, regulate the bone remodeling	2.Reduce the DNA binding affinity of a transcription factor c-jun AP-1 and NF-κB	Tofino-Vian et al., 2018
	3.Promote the proliferation and migration of OA chondrocytes, maintain the chondrocyte matrix	3.Increase COL-II synthesis and decrease MMP-1, MMP-3, MMP-13, and ADAMTS-5 expression, inhibit the infiltration of M1 macrophages	Woo et al., 2020
	4.Enhance the proliferation and chondrogenic potential of periosteal cells	4.Upregulate miR-145 and miR-221, protect chondrocytes from H_2_O_2_-induced apoptosis	Zhao et al., 2020
	5.Suppress matrix degradation, promote matrix synthesis, reverse IL-1β-induced apoptosis	5.MiR-100-5p-regulated inhibition of mTOR-autophagy pathway	Wu et al., 2019
*BM-MSCs-Exos*	1.Reduce the level of inflammation, promote cartilage repair	1.Reinduce the expression of ACAN, COL-II, inhibit MMP-13, ADAMTS-5, and iNOS	Cosenza et al., 2017
	2.Inhibit the adverse effects of inflammatory mediators on cartilage homeostasis	2.Abrogate the upregulation of COX2 and interleukins, inhibit collagenase activity	Vonk et al., 2018
	3.Restore chondrocyte vitality	3.Inhibit the phosphorylation of p38 and ERK and promote the phosphorylation of AKT	Qi et al., 2019
	4.Promote cartilage repair and ECM synthesis, alleviate knee pain in OA	4.Alleviate the upregulation of CGRP and iNOS in the DRG tissue	He et al., 2020a
	5.Relieve pain, attenuated cartilage degeneration, facilitate subchondral bone remodeling	5.Inhibit TRAP expression and RANKL- RANK-TRAF6 signaling activation	Li J. et al., 2018
	6.Relieve chondrocyte damage in OA	6. Exosomal miR-127-3p inhibits CDH11 in chondrocytes, thereby blocking the Wnt/β-catenin pathway activation	Dong et al., 2021
	7.Stimulate migration and proliferation of chondrocytes	7.Regulate chondrocyte formation through BMP4 signaling	Zhou X. et al., 2020
	8.Alleviate inflammation and OA-like damage	8.Exosomal miR-9-5p downregulates the level of inflammatory factors and reduces oxidative stress injury by regulating SDC1	Jin et al., 2020a
	9. Promote chondrocyte migration and inhibit cartilage degeneration	9. Exosomal miR-136-5p targets ELF3	Chen et al., 2020
*SMMSCs-Exos*	Suppress the inflammatory response and apoptosis of chondrocytes	Exosomal miR-129-5p targets the 3'UTR end of HMGB1 and inhibits IL-1β-mediated upregulation of HMGB1	Qiu et al., 2021
*UC-MSCs-Exos*	1.Promote chondrocyte proliferation, migration and matrix synthesis, and inhibit apoptosis	1.The activation of TGF-β 1 and Smad2/3 signaling	Yan and Wu, 2020
	2.Promoted the migration, proliferation, and differentiation of chondrocytes and hBMSCs	2.Exosomal miR-23a-3p activates PTEN/AKT signaling	Hu et al., 2020
	3.Promote the migration and proliferation of BMSCs and the proliferation of chondrocytes	3.Promote the polarization of macrophages toward the M2 phenotype and inhibit the inflammatory response	Jiang et al., 2021
*AFSC-Exos*	Enhance pain tolerance and induce an almost complete restoration of hyaline cartilage with good surface regularity	Inhibit M1 polarization	Zavatti et al., 2020
*CC-Exos*	1.Increase collagen deposition and minimize vascular ingrowth	1.Stimulate CPCs proliferation and increase expression of chondrogenesis markers while inhibit angiogenesis	Chen et al., 2018
	2.Promote chondrogenic differentiation of BMSCs	2.Exosomal miR-8485 targets GSK3B to repress GSK-3b expression and targets DACT1 to induce Ser9, activating Wnt/β-catenin pathways	Li et al., 2020
*D0-Exos*	Prevent the development of OA	Restore mitochondrial dysfunction and polarize macrophage response toward an M2 phenotype	Zheng et al., 2019
*TSCs-Exos*	1.Balance tendon ECM and promote the tenogenesis of TSCs	1.Increase the expression of TIMP-3 and COL-1a1	Wang et al., 2019
	2.Tendon repair	2.Induce tenogenic differentiation of MSCs in a TGF-β-dependent manner	Xu et al., 2019
*SMSCs-140-Exos*	Enhance the proliferation and migration of ACs without damaging ECM secretion	Suppress the expression of RalA, hence reversing the inhibition of SOX9	Tao et al., 2017
*SMSC-155-5p-Exos*	Promote proliferation and migration, suppress apoptosis and enhance ECM secretion of OA chondrocytes	Target RUNX2	Wang et al., 2021
*hMSCs-miR-92a-3p-Exos*	Promote chondrogenesis and inhibit cartilage degradation	Upregulate the expressions of COL2A1, COL9A1, ACAN, SOX9, and COMP, downregulate the expressions of COL10A1, RUNX2, and MMP-13	Mao et al., 2018b
*AC-miR-95-5p-Exos*	Facilitate matrix expression and enhance cartilage formation	Suppress the activity of reporter constructs containing the 3'-UTR of HDAC2/8	Mao et al., 2018a
*hBMSC-320c-Exos*	Increase the proliferation and migration of chondrocytes, enhance the chondrogenic potential of BMSCs	Downregulate MMP-13 and upregulate SOX9 expression	Sun et al., 2019
*hBMSC-miR-26a-5p-Exos*	Alleviate the damage of synovial fibroblasts	Target PTGS2	Jin et al., 2020b
*BMSCs-210-Exos*	Improve the proliferation of chondrocytes and inhibit LPS-induced cell apoptosis	Inhibit the expression of Tnfrsf21 and attenuate NF-κB pathway	He et al., 2020b
*MSC^*KLF*3−*AS*1^-Exos*	Induce proliferation and inhibit apoptosis	*Via* miR-206/GIT1 axis	Liu et al., 2018
*TGF-β1-Exos*	1.Promote chondrocyte proliferation and cartilage repair	1.Downregulate Sp1	Wang et al., 2018
	2.Promote M2 polarization of synovial macrophages	2.Target MAPK6	Wang and Xu, 2021
*MSC-IL-Exos*	Enhance anti-inflammatory activity in osteoarthritic SW982 cells	MiR-147b-mediated inhibition of the NF-κB pathway	Kim et al., 2021
*KGN-BMSC-Exos*	Endow BMSC-Exos with stronger chondral matrix formation and less degradation	Avoid the depression of RUNX1 in BMSC-Exos treated chondrocytes	Liu et al., 2020

The ability of EVs in diagnosing joint diseases has been initially revealed. As such, the rise of synovial miR-200C and the decline of plasma miR-132 contribute to the diagnosis of OA. Besides, the severity of OA, which is considered as an important reference to undergo a surgical treatment of arthroplasty, can also be assessed by the degree of elevating synovial LncRNA PCGEM1 and decreasing plasma let-7e. However, the accuracy and sensitivity of EVs as a diagnostic tool for joint diseases need to be confirmed by more experimental and clinical studies. Furthermore, current detection methods of EVs are time-consuming and complex, which limit the usage of EVs as a routine clinical testing, thus the most accurate and convenient EVs indicators are still being explored.

Urine, another fluid sample rich in EVs, can be obtained in a less invasive manner and easier to manipulate and store compared to blood and synovial membranes (Merchant et al., 2017). It has been widely recognized that urine EVs could reflect the physiological status of the urinary system and serve as the biomarkers of kidney disease. Interestingly, Viñuela-Berni et al. found a high percentage of CD14+, CD3+, and CD19+ in urine EVs of patients with active rheumatoid arthritis (Viñuela-Berni et al., 2015), which opened up the revenue for the use of urine EVs in the diagnosis of joint diseases.

Extracellular vesicle-based therapies hold a great promise; however, there are still many issues that need to be addressed for their direct use in the clinical treatment of OA. One of the major challenges is a low retention rate. Now the administration of exosomes in most animal experiments was a direct injection of the pre-prepared exosome-containing suspension into the joint cavity. Due to liquid dissipation, exosomes could not be maintained at the injury site for a long period of time, and the injection had to be repeated regularly, which increased the complexity of the experimental operation and the risk of intra-articular infection (Kim Y. G. et al., 2020). For this reason, some scholars proposed to integrate exosomes into biomaterials to assemble tissue engineering chimeras with a sustained release function so as to prolong the retention time of exosomes at the lesion site and to play a long-term therapeutic role. Liu et al. (Figure 5C) integrated SC-Exos with a photoinduced imine crosslinking hydrogel glue as an exosome scaffold to prepare an acellular tissue patch [electrohysterography (EHG)], and placed it at the cartilage injury site (Liu et al., 2017). The results showed that EHG could retain SC-Exos *in vitro*, combine with a cartilage matrix, and promote the deposition of cells in the lesion, further leading to cartilage repair. Noteworthy, the greatest advantage of hydrogel is after being injected into the recipient site, it could set by gelation to fill cartilage defects of any shape and size. Chen et al. designed a biological scaffold (cartilage ECM/gelatin methacrylate/exosome scaffold) for the MSC-Exos delivery by three-dimensional (3D) printing technology, and evaluated its repair ability through a rabbit osteochondral defect model (Chen et al., 2019). The results suggested that this scaffold could effectively restore the mitochondrial dysfunction of chondrocytes and significantly promote cartilage regeneration. Although the introduction of biomaterials can improve the retention rate, the biocompatibility and biodegradability need to be fully considered prior to the clinical translation.

Another challenge in the EV-based therapy lies in the method of harvesting large-scale exosomes with a consistent cargo. Although large-scale bioreactors have been developed to culture cells, in a bid to harvest a sufficient number of exosomes for downstream clinical applications, the purification and isolation process are still labor-intensive and unnecessarily increase the production cost, impeding the success of downstream commercialization. Additionally, in order to improve the therapeutic effect and consistency of the cargo, current studies mostly focus on the loading of specific miRNAs into exosomes by modifying the exosome-secreting cells with transfection or integrating exosomes into biological scaffolds to prolong the functional time of the drugs at desired dosages. However, the natural articular cartilage is formed through the cell-to-cell interaction, growth factors, and appropriate mechanical stimulation in the absence of scaffolds and other carriers. A key research direction is on the ways to simulate the microenvironment in the process of cartilage development so as to induce MSCs to differentiate into chondrocytes and form a multilayer cartilage structure similar to natural cartilage. Moreover, the standardized dosage, pharmacokinetics, and biodistribution of exosomes need to be further explored and verified in long-term and extensive experimental studies.

Current studies have been heavily focused on the exosome-based therapy despite the fact that MVs may potentially possess the similar or even a better therapeutic effect for cartilage repair due to their improved anti-inflammatory capacity. Hence, future studies should also look into the role of MVs during the pathological alteration and also their potentials as a diagnostic and therapeutic tool for joint diseases.

With the deepening of studies on the mechanism of EVs in tissue repair and regeneration and on updating the methods of research, we reasonably believe that EVs play a principal role in the diagnosis and treatment of ACD and OA and is extensively used in joint diseases.

## Author Contributions

XC and KT participated in the design of a schematic diagram of EVs. All authors participated in manuscript preparation.

## Conflict of Interest

The authors declare that the research was conducted in the absence of any commercial or financial relationships that could be construed as a potential conflict of interest.

## Publisher's Note

All claims expressed in this article are solely those of the authors and do not necessarily represent those of their affiliated organizations, or those of the publisher, the editors and the reviewers. Any product that may be evaluated in this article, or claim that may be made by its manufacturer, is not guaranteed or endorsed by the publisher.
